# Bioinspired and Multifunctional Tribological Materials for Sliding, Erosive, Machining, and Energy-Absorbing Conditions: A Review

**DOI:** 10.3390/biomimetics9040209

**Published:** 2024-03-30

**Authors:** Rahul Kumar, Mansoureh Rezapourian, Ramin Rahmani, Himanshu S. Maurya, Nikhil Kamboj, Irina Hussainova

**Affiliations:** 1 Department of Mechanical and Industrial Engineering, Tallinn University of Technology, Ehitajate Tee 5, 19086 Tallinn, Estonia; mansoureh.rezapourianghahfarokhi@taltech.ee (M.R.); himanshu.singh.maurya@associated.ltu.se (H.S.M.); nikhil.kamboj@utu.fi (N.K.); irina.hussainova@taltech.ee (I.H.); 2 CiTin–Centro de Interface Tecnológico Industrial, 4970-786 Arcos de Valdevez, Portugal; ramin.rahmaniahranjani@gmail.com; 3 proMetheus–Instituto Politécnico de Viana do Castelo (IPVC), 4900-347 Viana do Castelo, Portugal; 4 Department of Engineering Sciences and Mathematics, Luleå University of Technology, 97187 Luleå, Sweden; 5 Department of Mechanical and Materials Engineering, University of Turku, 20500 Turku, Finland; 6 TCBC–Turku Clinical Biomaterials Centre, Department of Biomaterials Science, Faculty of Medicine, Institute of Dentistry, University of Turku, 20014 Turku, Finland

**Keywords:** bionic surface texturing, sliding wear, erosion, dynamic impact, cutting tools, friction, biomaterials, mechanical property, surface modification, biomimetics, nature-inspired tribology, sustainable development, green tribology, metamaterials

## Abstract

Friction, wear, and the consequent energy dissipation pose significant challenges in systems with moving components, spanning various domains, including nanoelectromechanical systems (NEMS/MEMS) and bio-MEMS (microrobots), hip prostheses (biomaterials), offshore wind and hydro turbines, space vehicles, solar mirrors for photovoltaics, triboelectric generators, etc. Nature-inspired bionic surfaces offer valuable examples of effective texturing strategies, encompassing various geometric and topological approaches tailored to mitigate frictional effects and related functionalities in various scenarios. By employing biomimetic surface modifications, for example, roughness tailoring, multifunctionality of the system can be generated to efficiently reduce friction and wear, enhance load-bearing capacity, improve self-adaptiveness in different environments, improve chemical interactions, facilitate biological interactions, etc. However, the full potential of bioinspired texturing remains untapped due to the limited mechanistic understanding of functional aspects in tribological/biotribological settings. The current review extends to surface engineering and provides a comprehensive and critical assessment of bioinspired texturing that exhibits sustainable synergy between tribology and biology. The successful evolving examples from nature for surface/tribological solutions that can efficiently solve complex tribological problems in both dry and lubricated contact situations are comprehensively discussed. The review encompasses four major wear conditions: sliding, solid-particle erosion, machining or cutting, and impact (energy absorbing). Furthermore, it explores how topographies and their design parameters can provide tailored responses (multifunctionality) under specified tribological conditions. Additionally, an interdisciplinary perspective on the future potential of bioinspired materials and structures with enhanced wear resistance is presented.

## 1. Introduction

**Figure 1:**
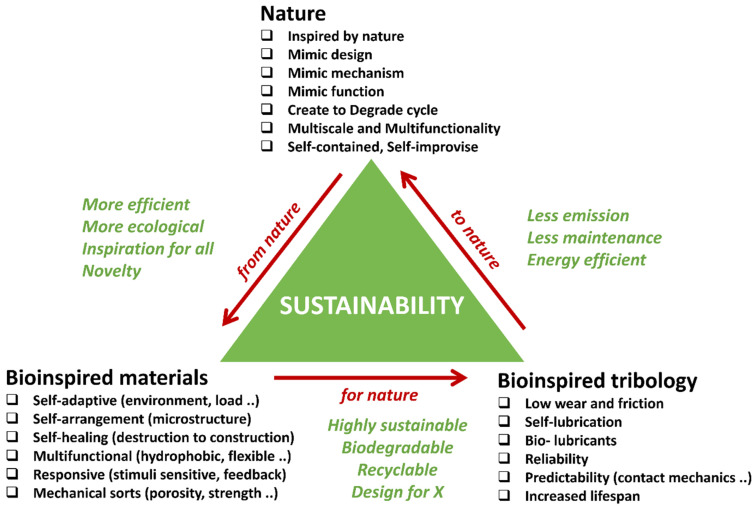
Sustainability triangle showing nature, materials, and tribology as its corners.

Sustainable development is closely linked to improvement in tribology (low friction, low wear, and enhanced lubrication), which has a significant and beneficial impact on both the environment and society [1]. Global energy consumption is increasing year on year, putting pressure on both resources and the environment. Notably, the repercussions of friction and wear extend beyond substantial energy and economic loss (approximately 23% of global energy loss and 1–2% of a nation’s GDP) and serve as a major source of CO_2_ emissions [2,3]. Crucially, tribological losses (material degradation), comprising high friction and wear inefficiencies, expenses related to part replacement and remanufacturing, and maintenance costs, affect virtually all moving elements, ranging from industrial machinery to the intricate mechanics of natural bone joints and artificial implants integrated into the human body [4]. Any effort aimed at curbing these losses yields a direct and positive impact, not only conserving energy but also fostering economic stability, and enhancing the well-being of both individuals and the environment. Consequently, it is entirely valid to assert that tribology, with its inherent capability to diminish friction and wear, characteristically contributes to the noble pursuit of a sustainable and human-centric world, and directly or indirectly contributes to Sustainable Development Goals 3, 6, 7, 8, 9, 11, 13, 14, and 15 [5]. Figure 1 shows the triangle of sustainability, revealing nature, bioinspired materials, and bioinspired tribology as its three corners.

In this context, various methods can mitigate friction and its negative effects, such as wear. Those can be classified into (i) the mechanical approach (e.g., well-lubricated systems, novel composites, microstructural enhancements, and heat treatments, etc.) and (ii) the surface topography engineering approach (e.g., adjusting surface roughness or texture to reduce contact area). According to a model proposed by McFarlane and Tabor [6], friction force is the sum of two components: a mechanical contribution resulting from surface deformation and an adhesive contribution mainly influenced by surface energy. This model suggests that a minimum coefficient of friction (CoF) can be achieved at a critical value of real contact area [7]. Consequently, controlling surface topography can lead to CoF control. However, traditionally, the focus has been on mechanical approaches, overlooking the potential of engineered interfaces in friction control due to incomplete understanding of surface texturing (optimal choice and design of textures). Additionally, an innovative approach utilizes a combination of both approaches to enhance tribological properties and reduce friction and wear. The synergy may enable simultaneous tribological benefits, such as favorable effects of wear debris, continuous lubricant replenishment, formation of protective tribolayers or tribofilms, etc. [8,9].

Multifunctionality within tribological materials/surfaces represents a paradigm shift in engineering [10]. These materials not only mitigate wear and friction but also offer measurable benefits for efficient operation in specific applications. For instance, the wear rate of modern hip implants has been reduced by several orders of magnitude [11]. However, to enhance their longevity and minimize the necessity for revision surgeries, it is equally crucial for biomaterials to positively carry dynamic fluctuating high loads (during human movement), facilitate cell transfer and growth (biological interaction), and ensure seamless tribological integration within the human body (chemical interaction), even leveraging the positive effects of wear debris (e.g., hydroxyapatite-based biomaterials can generate wear debris that stimulates bone growth and integration around the implant, while optimized surface roughness/texture can lead to improved osteoconductivity or lubrication) [11,12,13,14]. Similarly, in the domain of photovoltaic (PV) solar cells, materials/surfaces are engineered to reduce wear (erosion) while simultaneously improving light absorption and self-cleaning properties [15]. This innovation can result in remarkable, up to 15%, enhancement in the overall efficiency and lifespan of solar panels [16]. In hydro-bearings and hydrofoils, meticulously designed surface textures that reduce drag lead to elevated energy conversion rates [17,18]. In certain instances, these materials have demonstrated substantial efficiency gains exceeding 20%, thereby translating into increased renewable energy production. In other cases, such as surgical instruments or soft robotics, it is advisable that the surface demonstrate higher friction or higher adhesion in order to improve the grip strength while accommodating high load transfer (pick up/drop) [19,20]. In space shuttles and satellites, the surfaces are designed to resist impact (wear) from space debris (meteors) while being lightweight, anti-weathering, etc. [21,22,23]. Space rover wheels are designed to navigate easily through harsh terrain, requiring enough flexibility while delivering tribological robustness [24]. Highly durable surfaces that demonstrate water-repellent properties, efficient water harvesting and spreading, or exceptional resistance to oils play a pivotal role in various applications [25,26,27]. These surfaces are essential for ensuring rapid lubricant dispersion, enabling rainwater harvesting systems, and powering nanogenerators, among other uses [28]. For instance, in the context of lubrication, surfaces with superoleophobic characteristics facilitate swift and uniform distribution of lubricants in machinery, reducing friction and wear. Moreover, surfaces engineered for efficient water spreading enable collection and storage of rainwater for various purposes while being resistant to enduring impact, erosion, etc. In summary, the creation of wear-resistant surfaces with specific functionalities, such as water harvesting and superoleophobic properties, has far-reaching implications across a range of applications, from industrial machinery to sustainable water management and energy harvesting technologies [29]. Frictional anisotropy is a critical tribological feature that spans from the molecular scale to the macroscale, characterized by directional asymmetry in friction response during sliding [30]. This feature is particularly valuable for microbots designed for friction-based locomotion, offering high maneuverability and precise targeting in confined three-dimensional (3D) spaces. Additionally, it ensures stress compatibility with soft living matter by limiting interface stresses [31]. As the demand grows for microbots in various applications like robotic assembly, drug delivery, lab-on-chip technology, sensing, microsurgery, and cancer treatment, there is a practical need to construct artificial prototypes [32]. These prototypes, exploiting geometric features to induce controllable anisotropic motion responses under external oscillating loads, serve as models for understanding and predicting the dynamics of future industrial microactuators [33]. For instance, in medical microbots navigating complex biological environments, anisotropic friction-based locomotion coupled with physical propulsion holds promise as an enabling technology for their development [34]. Table 1 shows the essential functional features required in key tribologically challenged fields.

Recently, significant attention has been paid to the exploration of materials, surfaces, and architectures inspired by nature [25,26,62]. The fact that natural objects have survived the harshest conditions through multifaceted evolution inspires engineers to create or imitate the construction of similar structures. Nature-inspired materials are man-made materials that mimic the structure, properties, or functions of natural materials or living organisms and offer the potential for sustainable synergy between tribology and multifunctionality [63]. The properties of these materials and surfaces result from complex interplay between the surface structure and the morphology and physical and chemical properties. Moreover, many materials, surfaces, and devices with such designs provide multifunctionality. Various terms such as bioinspiration, biomimicry, biomimetics, nature inspiration, and nature mimicry are commonly used interchangeably by researchers [63]. Biomimetic materials imitate the evolutionarily developed structural features, leading to adapted architecture, especially in living species [64,65,66]. Often, the architectures exhibit a graded structure spanning multiple scales, including macro-, micro-, and nanoscales. For example, bone’s complex porous structure, characterized by complex ligament shapes and variations in density, allows it to achieve superior mechanical (stiffness, energy absorption, stress distribution, etc.) and biological characteristics (e.g., porosity leads to facilitating nutrient exchange and cell proliferation) compared to most man-made materials (biomaterials) [65].

**Figure 2:**
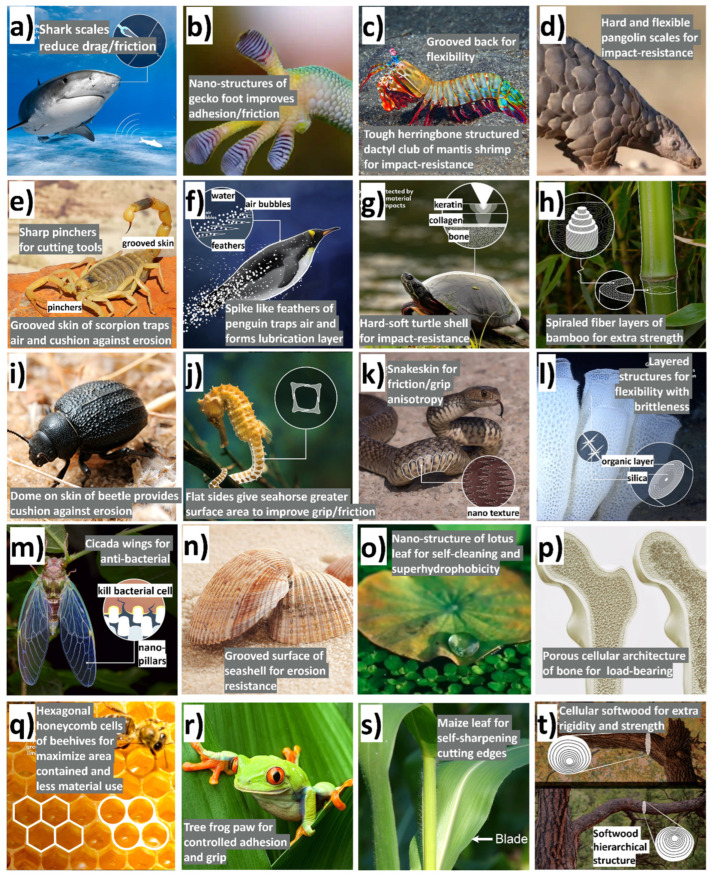
Bioinspired (nature) inspiration influencing various tribological conditions: (**a**) drag reduction in shark skin; (**b**) adhesion switch in gecko foot; (**c**) club of mantis shrimp; (**d**) flexibility of pangolin scales; (**e**) scorpion back; (**f**) drag reduction in penguin skin; (**g**) turtle shell; (**h**) fibered hierarchical layer of bamboo; (**i**) dung beetle back; (**j**) adhesion shift in sea horse tail; (**k**) friction anisotropy in snakeskin; (**l**) layered structure of Venus flower basket; (**m**) antibacterial cicada wings; (**n**) seashell; (**o**) hydrophobicity of lotus leaf; (**p**) trabecular bone network; (**q**) honeycomb cells; (**r**) adhesion manipulation in tree frog paw; (**s**) self-sharpening of maize leaf; and (**t**) hierarchical structure of softwood. Images reproduced with permission from The Biomimicry Institute, USA [91]. Reported areas of desired functionality concerning tribology are mentioned.

Friction and adhesion are common in nature. Notable examples involve leveraging nature-inspired surface textures found in animal scales and skins, such as those of sharks (for drag reduction and hydrophobicity), snakes (for erosion resistance), pangolins and turtles (for flexibility combined with erosion/abrasion resistance), and gecko feet (for improved adhesion) [24,63,67]. Some species feature adaptive systems that enable changes in color, pattern, or texture for defense, signaling, temperature regulation, or reproduction (also called the ‘chameleon mimetic system’) [68,69]. Nacre, an organic material found in mollusk shells, exhibits remarkable strength and resilience [70]. Spider silk, renowned for its exceptional mechanical properties and supercontraction abilities, holds great potential for various structural applications [71]. Examining beetle adhesion systems at the nanoscale has uncovered a diverse array of intricate multiscale architectures serving crucial functions like wing fixation, crawling, mating, and external protection, mainly utilized regarding locomotion for microrobots (anisotropic friction and earthworm-inspired) [30,34,72]. These adhesion systems utilize different mechanisms; some rely on liquid secretion (capillary force and lubrication), while others operate through direct interlocking of high-density microfibers or contact of mushroom-shaped hairy structures (van der Waals force) [70]. A comprehensive analysis of materials (structures) inspired by nature reveals their unique functionality, encompassing various domains of sustainable science [63]. Bioinspired structures combined with surface topography (or texturing) of these materials demonstrate promising outcomes in customizing friction, wear, and other captivating properties such as antifouling [73,74,75], self-lubrication [8,61,76], self-adaptation [77,78], hydrophobicity [27], self-cleaning [16,25,73], cell growth [12,79], drug delivery [80,81,82,83,84,85,86], antibacterial [79,87,88], color manipulation [68], anti-reflection [89], anisotropic friction in MEMS/NEMS/microrobots/microactuators [30,70,72], and enhanced adhesion [24,25,70,90]. Figure 2 shows various biological organisms (animals/plants) for inspiration regarding creating an efficient multifunctional tribological material.

Achieving such complex nature-inspired geometries in materials or on their surface through conventional materials and technologies often proves challenging and expensive [92,93,94,95]. Historically, progress regarding materials (tribological) has primarily depended on modifying their chemical composition to alter their tribological and mechanical properties. Although this approach has yielded positive results, the journey from discovering new tribomaterial to its commercial availability has typically been time-consuming [92]. To this end, additive manufacturing (3D printing or laser surface texturing) offers promising prospects, especially in terms of generating complex bioinspired surfaces/materials and also allowing for faster fabrication and up-scaling production [94,95,96,97,98,99].

**Figure 3:**
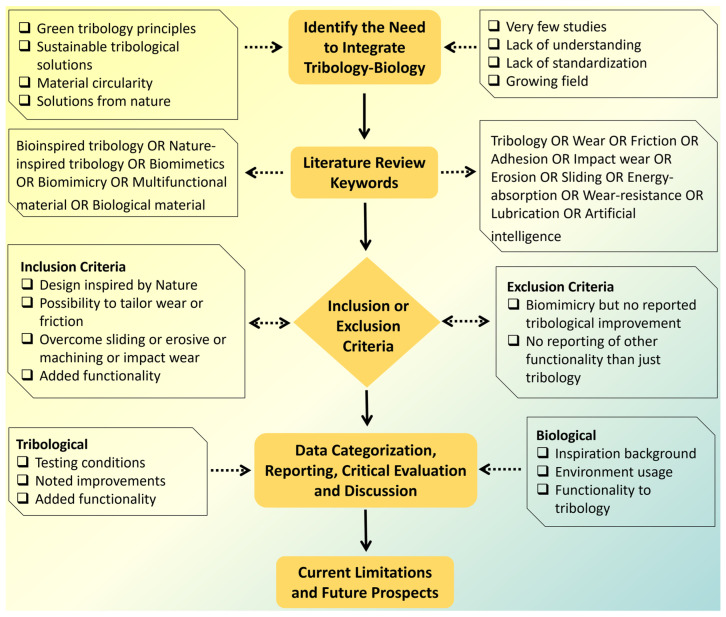
Research methodology framework. The arrow directions signify the flow direction of data.

Any bottom-up approach to developing complex, multifunctional, and multiscale mimetics that can provide multiple robust functionalities requires a multidimensional strategy. The research in this multifaceted tribology domain is still at an early stage. The purpose of this review is to provide comprehensive information on the latest advances and future prospects in the field of “nature inspired” or “biologically inspired” tribological materials, with particular emphasis on their design, manufacturing processes, sources of inspiration, as well as friction and wear, performance, and other additional functions. The key focus is to establish a correlation between various tribological scenarios, bioinspired material characteristics, and their resilience to specific environmental conditions, aiming to formulate guidelines for the creation of bioinspired wear-resistant materials and systems. We have attempted to sum up relevant tribological information to a large extent. Nevertheless, due to some missing parameter specifications from the relevant reference publications that do not clearly and unambiguously characterize the physical conditions or tribological stress or the fact that tribological behavior is a system response and mere representation of some value such as force, rotational frequencies, etc., it is not in itself (alone) suitable for characterizing the stress level of the tribological system in question. Also, mixed or incorrect use of data/units for mass and forces should be avoided or carefully analyzed. For this, we recommend that readers defer to the relevant cited source(s). Figure 3 illustrates the research methodology framework for the current work.

## 2. Design and Fabrication Strategies for Bioinspired Tribological Surfaces

**Figure 4:**
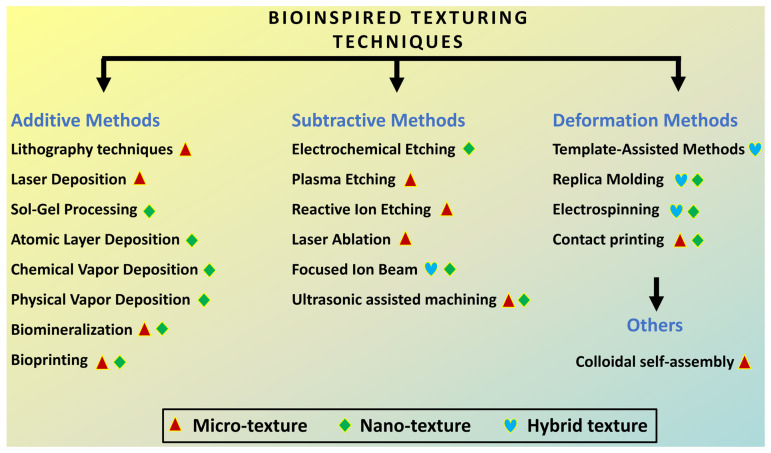
Bioinspired texturing techniques based on scales of textures and methods of fabrication.

Bioinspired texturing techniques encompass a range of methods that mimic natural surface structures found in various biological systems. These techniques can be broadly classified into additive, subtractive, and deformation methods, each offering unique capabilities for creating surface textures with tailored properties and functionalities, benefiting a wide range of applications in tribology, biomedicine, materials science, and beyond. Figure 4 illustrates key texturing techniques as reported in the literature based on scales of textures and methods of fabrication.

Additive methods involve building up material to form desired textures. Lithography techniques, such as photolithography and electron-beam lithography, utilize masks or focused beams of light/electrons to selectively expose or remove material, creating precise patterns on surfaces [100,101,102]. Examples include microfluidic channels for lab-on-a-chip devices and photonic structures for optical applications.

Laser deposition involves using lasers to melt and deposit material onto a substrate, forming intricate structures layer by layer [97]. This technique is commonly used in additive manufacturing processes, including selective laser sintering and direct laser writing, to create complex 3D geometries with high resolution and accuracy [103].

Sol–gel processing involves the conversion of precursor solutions into solid materials through hydrolysis and condensation reactions [104,105]. By controlling the composition and processing conditions, sol–gel techniques can produce thin films, coatings, or bulk materials with tailored microstructures and properties. Applications include optical coatings, protective coatings for corrosion resistance, and biomedical implants.

Bioprinting utilizes CAD models to deposit biological materials, such as cells, growth factors, and biomaterials, layer by layer to create 3D structures. This technique is used in tissue engineering and regenerative medicine to fabricate customized scaffolds for tissue repair and organ transplantation [106,107].

Subtractive methods involve removing material to create textures on surfaces. Electrochemical etching utilizes electrochemical reactions to selectively dissolve material from a substrate, creating microscale or nanoscale features with high aspect ratios. This technique is widely used in semiconductor fabrication to pattern silicon wafers for microelectronics and MEMS devices [108].

Plasma etching involves using reactive plasma gases to remove material chemically or physically from a substrate, creating precise patterns through a process known as dry etching [108]. This technique is used in microfabrication processes to pattern semiconductor materials, polymers, and metals for electronic devices, sensors, and optical components.

Laser ablation utilizes high-power laser pulses to vaporize and remove material from a substrate, creating microscale or nanoscale features with high precision and resolution. This technique is used in materials processing, microfabrication, and surface modification applications, including micromachining, engraving, and thin-film patterning [84,109,110].

Ultrasonic-supported surface texturing is a specialized machining technique that utilizes ultrasonic vibrations to enhance the cutting action of the tool during surface texturing operations [111]. In this process, ultrasonic vibrations are applied to the cutting tool or workpiece, causing rapid small-scale movements that help to break up chips and improve material removal efficiency. This technique is particularly useful for creating microscale or nanoscale surface textures with precise control over texture depth, shape, and orientation.

Deformation methods alter surface textures through mechanical manipulation. Template-assisted methods involve using templates or molds with predefined patterns to transfer textures onto substrates through techniques such as nanoimprint lithography and soft lithography. Replica molding utilizes elastomeric molds to replicate surface textures from master molds onto different substrates, enabling large-scale production of biomimetic surfaces for various applications [34,100,112,113].

Electrospinning involves using an electric field to draw and elongate polymer solutions or melts into thin fibers, which are deposited onto a substrate to form textured surfaces with controlled fiber orientations and dimensions [114]. This technique is used in tissue engineering, filtration membranes, and functional textiles [115].

Contact printing utilizes patterned stamps or molds to transfer ink or functional materials onto substrates, creating textured surfaces with micrometer-scale or nanometer-scale features [116]. This technique is used in flexible electronics, sensors, and microfluidic devices for patterning conductive materials, biomolecules, and functional coatings.

Colloidal self-assembly involves the spontaneous organization of colloidal particles into ordered structures on a substrate through processes such as sedimentation, evaporation, or chemical interactions [117]. This technique is used in nanotechnology, photonic crystals, and surface engineering to create hierarchical structures with controlled properties for optical, electronic, and sensing applications.

## 3. Bioinspired Surfaces for Sliding Contacts

Sliding contacts bionic modifications and the bionic tribological surfaces thereof are designed to optimize lubrication (solid or liquid) and capture wear debris, such as in processes like honing cylinder liners. By creating bionic textures with controlled geometry, these surfaces retain lubricating oil and trap wear debris generated during rubbing. The material volume between the bionic textures acts as raised islands, cushioning the counterbody and reducing the actual contact area, thereby improving friction quality, and sometimes improving flexibility to accommodate sudden jerks. Properly structured cushioning islands can also reduce wear. The biotexturizing process often aims to adjust lubricating fluid film thickness, facilitating early separation between rubbing surfaces upon lubricant entry and reducing friction over a shorter distance [118]. It has been shown that biotextures can contribute to wear reduction by means of (i) enhancing hydrodynamic pressure to improve load carrying capacity and decrease shear–strain rate in the lubricant (hydrodynamic bearing effect), (ii) attracting more lubricant into the contact area (inlet suction effect), (iii) serving as a reservoir to store and supply lubricant to the interface (lubricant reservoir effect), (iv) capturing wear particles (debris-trapping effect), and (v) decreasing the actual contact area, thereby reducing friction [119,120]. It must be noted that Ra values (roughness) are commonly reported for surface texture analysis in most of the published work. However, it is important to note that Ra, owing to its double averaging, does not inherently convey significant information.

**Figure 5:**
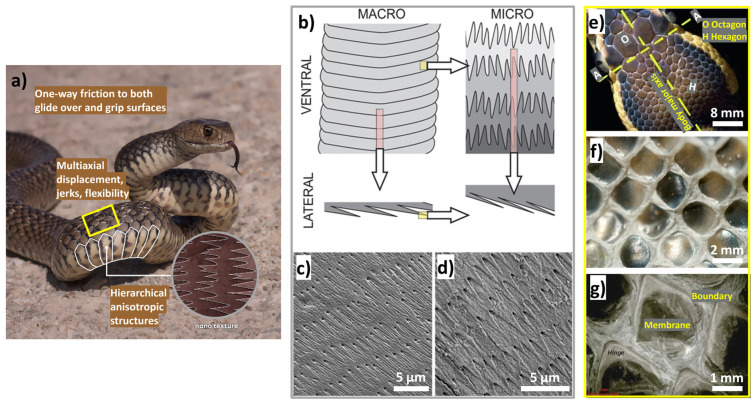
Images showing (**a**) snake slithering mechanism. Adopted from The Biomimicry Institute, USA [91]; (**b**) hierarchical anisotropic structures on the ventral snake surface [122]; (**c**) SEM of western diamondback rattlesnake [122]; (**d**) SEM of black-necked spitting python [122]; (**e**) details of dorsal (back) scale structure of a python [121]; and (**f**,**g**) magnified image of a python dorsal [121].

Additionally, certain bionic textures such as snake or shark skin/scales, maize leaf, etc., are known to exhibit frictional anisotropy, adding to their unique features. For example, in Figure 5, a typical snake slithering mechanism reveals varying topography across different areas of the snake’s skin. Pythons, known for constricting their prey before ingestion, must accommodate the prey’s volume and shape by stretching their jaws upon inhalation. This process can result in multi-axial displacements of the skin’s surface material [121]. To address this, the skin above line AA is composed of small patches of uniform octagonal shape connected by flexible strips. This design enables multi-axis stretching of the surface while potentially damping sudden jerks caused by prey resistance, as observed in the throat region. However, the frictional properties of the ventral surface (that touches the ground during slithering) of snakeskin vary widely, covering a broad range of values. Additionally, the degree of anisotropy, or directionality of friction, also varies within this range [122,123,124,125,126]. However, Filippov et al. [122] conducted a study comparing frictional experiments with anesthetized snakes on smooth and rough surfaces (with roughness values of Ra  =  20 and 200 μm, respectively), revealing frictional anisotropy that diminished significantly on the smooth surface. They found that the macroscopic pattern of ventral scales contributes to frictional anisotropy at both macro- and microscales, with two hierarchical levels of structures, i.e., scales and denticles, playing a role. This explains why snakes experience decreased locomotory ability on smooth substrates and rely on a certain level of roughness for propulsion, in line with their numerical model’s predictions.

Mühlberger et al. [127] utilized a combination of powder injection molding and ultraviolet light-assisted nanoimprint lithography to replicate the intricate features of a Python/Cobra (*Naja nigrocollis*). This approach enabled the direct copying of the biological surface and imprinting of unit textural features three times the length of the actual features of the snake. Pin-on-disc experiments comparing the friction behavior of textured and polished ceramic samples showed favorable results for the textured surfaces. Snakeskin textured samples exhibited a 30% lower COF than polished non-textured samples, particularly when sliding in the parallel direction. However, the study emphasized the importance of employing an efficient replication method to achieve the required fine size texturization. Samples with features similar in length to the original skin did not perform well due to the inadequacy of the replication technique used rather than the tribological inefficiency of the geometric features themselves. In an another study by Greiner et al. [126], they used laser texturing to create scale-like morphologies on 100Cr6 bearing steel pins, inspired by snake and sandfish skin, for testing in both lubricated and unlubricated tribological contacts. The wide scale-like surface structure significantly reduced friction forces by over 40% in unlubricated conditions, while, in lubricated conditions, it increased friction by more than three times. In an another study, Kumar et al. [128] investigated the impact of lotus leaf-inspired dimple texturing (diameter 500 µm, depth 8 µm) on the dry sliding performance of bearing steel, yielding several notable findings. Two different types of dimple shapes were produced, namely circular- and bi-triangular-shaped dimples. Further, to study the effect of dimple density, two different types of arrangements in dimples were created, i.e., in radial and spiral arrays. It was observed that a spiral array has a higher and more stable number of microdimples than a radial array under the same contact area, so the density of dimples becomes important. Hence, a higher effective number of microcavities was observed in the contact zone for the spiral array compared to the radial pattern, leading to increased wear particle entrapment. Moreover, increasing dimple density from 7% to 20% enhanced wear debris entrapment, consequently reducing wear. However, once the cavity is filled, the texture effect diminishes. Thirdly, bi-triangular dimples with lower density were more effective than circular shapes in reducing both friction and wear, with 20% density exhibiting optimal performance. Finally, in dry conditions, the textured samples displayed lower coefficients of friction compared to the untextured ones. Several other studies conducted on lotus leaf-inspired dimpled surfaces point to its friction-diminishing character due to debris entrapment and lubricant storage effects [124,129,130,131,132,133].

**Figure 6:**
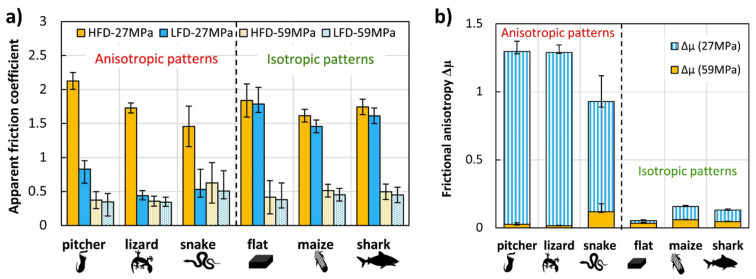
Images showing the progression of (**a**) CoF, i.e., µ, as function of the bioinspired surfaces for contact pressures and friction direction; and (**b**) direction-dependent CoF values of bioinspired surfaces for different contact pressure. The contact pressures were 27 MPa and 59 MPa. The friction directions are labeled as high friction direction (HFD) and low friction direction (LFD). Reproduced with permission from Ref. [134].

Han et al. [105] developed a novel bionic anti-adhesive electrode inspired by maize leaves, known for their anti-adhesive traits. The study investigated tissue adhesion effects on electrosurgical electrodes under high temperatures. Using the bionic anti-adhesive electrode, liver tissue was electrically cut in animal experiments. The results showed that the single bionic electrode had slightly better anti-adhesive properties, while the coupled electrode exhibited lower adhesion. The coupled electrode, coated with TiO_2_, gained superhydrophilic self-cleaning abilities. Later, Mzali et al. [134] studied the tribological behavior of five different surfaces’ patterns bioinspired from maize leaf skin, shark skin, snakeskin, pitcher’s structure, and lizard skin, with a focus on friction anisotropy and test conditions. Figure 6 depicts the apparent friction coefficient (CoF) evolution for various sliding cycles and contact pressures of 27 and 59 MPa [134]. Friction behavior significantly relies on surface texture and direction when interacting with anisotropic patterns. In the high-frictional direction (HFD), bioinspired patterns act as asperities, requiring substantial tangential load for deformation, correlating closely with plastic deformation and asperity sharpness. Asperities with sharper features induce higher plastic deformation, explaining the observed decrease in CoF (pitcher > lizard > snake). Conversely, in the low-frictional direction (LFD), the pattern effect is less pronounced, with an increase in pattern intensity resulting in higher absolute CoF values (0.8 to 0.4). When sliding in LFD, CoF decreases by 74%, 63%, and 61% for lizard, snake, and pitcher patterns, respectively. In contrast, isotropic patterns yield a large contact area (in relation to the apparent contact area), leading to relatively high CoF and increased adhesive wear. Increasing contact pressure reduces the friction coefficient, with a twofold pressure increase resulting in an 80% CoF drop. This phenomenon is attributed to frictional heat generation at the contact surface, affecting material strength and shear strength. In HFD, CoF ranges from 0.36 to 0.6, while, in LFD, CoF varies from 0.34 to 0.5. No significant difference in CoF between HFD and LFD is observed, indicating isotropic behavior among the tested specimens. However, at high contact pressures, flattening of surface asperities by the ball leads to early removal of high peaks, potentially reducing the microstructure’s impact on the friction coefficient. The anisotropic nature of friction evolution concerning bioinspired surface designs was reported by a few other authors [27,30,34,125,130,135,136].

**Figure 7:**
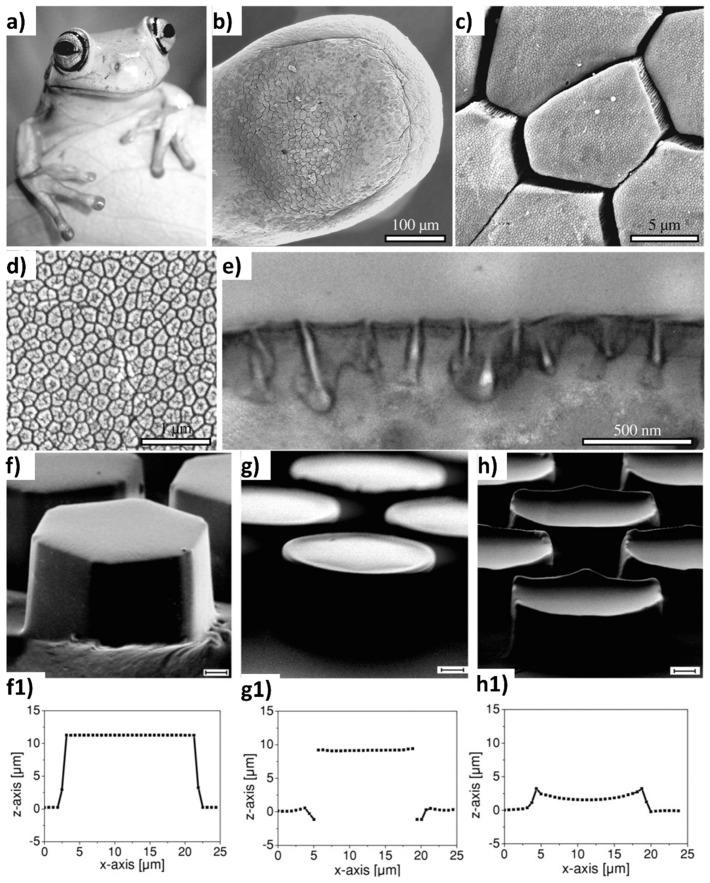
Morphology of tree frog toe pads. (**a**) White’s tree frog (Litoria caerulea); SEMs of (**b**) toe pad, (**c**) epidermis with hexagonal epithelial cells, and (**d**) magnified view of the surface of a single hexagonal cell showing peg-like projections; (**e**) TEM of cross-section through cell surface. Reproduced with permission from Ref. [141]. SEM images depict arrays of hexagonal micropillars on PDMS surfaces, with diameters ranging from 15 to 19 μm. These pillars feature various tip shapes, (**f**,**f1**) flat tips, (**g**,**g1**) T-shaped tips with heights of 10 μm and channel widths of 3 and 5 μm, as well as (**h**,**h1**) concave tips with a height of 3 μm and a channel width of 5 μm. Scale bars in the images correspond to 2 μm; (**f1**,**g1**,**h1**) corresponding profiles of the pillars obtained with a confocal microscope are provided below. Reproduced with permission from Ref. [140].

Furthermore, hexagonal textures exhibit noticeable anisotropy in various contact directions, influencing tribological properties. Nature’s diverse organisms, serving as models for texture design, showcase the remarkable effectiveness of hexagonal patterns. Many creatures feature hexagonal textures for specific functions: stability (e.g., insect compound eyes and honeycombs), adhesion (e.g., frog toes and clingfish), wear resistance (e.g., snake scales), lightweight structures (e.g., dragonfly wings), impact resistance (e.g., insect tentacles), and superhydrophobicity (e.g., bubble rafts and cicada toes), among others [137,138,139], for instance, the toe pads of tree frogs, characterized by hexagonal columnar epithelial cells separated by mucous-filled channels as shown in Figure 7 [140,141]. Tree frogs’ toe pads have garnered attention due to their ability to climb in humid environments without slipping. Similarly, hexagonal convex structures have been identified in the foot pads of katydids and bush crickets. Huang et al. and colleagues [132] engineered a surface structure inspired by tree frogs’ toe paw for wet sliding contacts, featuring elastic microdimple and -pillar patterns on polydimethylsiloxane elastomers (PDMS), to investigate its tribological performance. The friction behaviors of these patterned surfaces, lubricated with deionized water, were assessed. The results showed that the PDMS surface with microdimples exhibited reduced friction with increasing pattern area density. The area density of pillar patterns had a negligible impact on friction at low sliding speeds but became significant as sliding speed increased. The relationship between friction force and area density was approximately linear for pillar-patterned surfaces, possibly explaining the evolution of polygonal columnar structures in newts’ toe pads.

Lu et al. [142] conducted a study inspired by Seashell to design Ni_3_Al matrix bioinspired shell-like composite surface structures with excellent tribological properties. Wear tests were conducted to evaluate the effects of surface texturing and solid lubricants, revealing that the presence of solid lubricants significantly reduced friction coefficients and wear losses. The SAC305 alloy promoted self-compensating lubrication behaviors due to its ability to form a lubricating film under dry friction sliding. The combination of theoretical models and experimental measurements established a contact and collision model for microprotrusions between friction pairs, highlighting the roles of soft and hard materials in mutual sliding. The SAC305 alloy, with high surface energy and low hardness, facilitated material transfer and self-compensating lubrication compared to the Ni_3_Al matrix.

**Figure 8:**
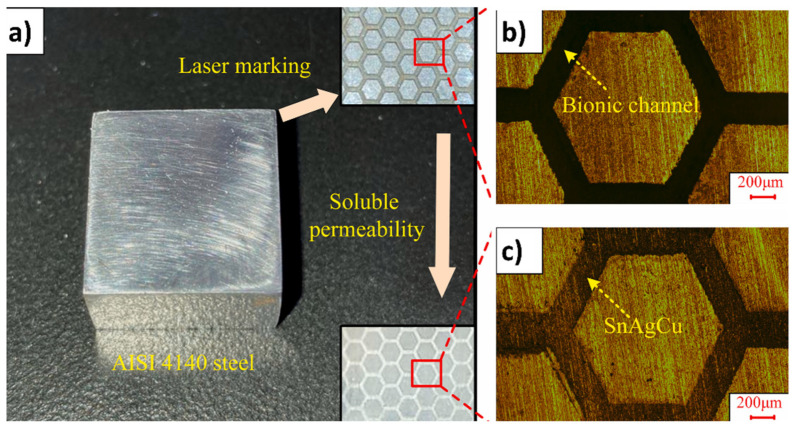
Showing the process of creating (**a**) AISI 4140 bionic self-lubricating material involves marking the specimen; (**b**) after laser processing and (**c**) then infiltrating it with solid lubricants. Reproduced with permission from Ref. [138].

In a separate investigation focusing on the combined effects of texturing and solid lubricants, researchers drew inspiration from the strong adsorption observed at the interface, known as the contact interface, which arises from the synergistic interaction between mucous (acting as a solid lubricant) and the surface microstructure of the frog paw, Huang et al. [137] investigated the tribological properties of AISI 4140 steel improved by biomimetic hexagonal textures (Figure 8) and incorporation of solid lubricants (Ag, Sn, and Cu) and TiC, and their optimization thereof [138]. Introducing TiC and SnAgCu showed significant antifriction and wear resistance. Compared to Ag and Sn, the modified material exhibited around a 65% decrease in average COF and around a 42% decrease in wear depth in addition to achieving a steady frictional condition (reduced fluctuations). The multi-solid lubricants formed a lubricating film and enabled self-repairing behavior for surface defects, contributing to excellent antifriction and wear resistance. Nano-TiC particles were gradually enveloped by ductile SnAgCu during wear, transitioning the contact mode from sliding to rolling for antifriction effects. Moreover, rolling lubrication particles were captured by wear defects, facilitating self-repair. In another study, Wang et al. [143] designed dung beetle-inspired multi-bioinspired hierarchical textures featuring superhydrophobicity to enhance AISI 440C steel wear resistance in water lubrication. It was shown that multi-bioinspired hierarchical textures significantly improved wear resistance under low loads and high frequencies (values are provided in Table 2). Superhydrophobic micro/nanostructures played a crucial role in enhancing wear resistance, resulting in wear reduction rates higher than single bioinspired textures. The geometrical parameters notably affected the wear properties, with the wear rates decreasing by 64% and 50% for textured configurations. The combined effects of multiple texturing and superhydrophobic micro/nanostructures boosted wear resistance by increasing surface hardness and improving hydrodynamic pressure. Several others have reported superior tribological performance (reduction in CoF/wear rate/wear mechanisms, etc.) upon synergistic application of biotexturing and solid lubrication [61,76,144,145]. These structures can trap lubricants (wear debris may lead to rolling friction), reduce contact area, improve hydrodynamic pressure, and alter the contact interface, leading to improved wear resistance, reduced friction, and contributing to increased efficiency, durability, and reliability of mechanical systems, making them valuable tools in various industrial applications.

## 4. Bioinspired Surface Texturing of Biomaterials

**Figure 9:**
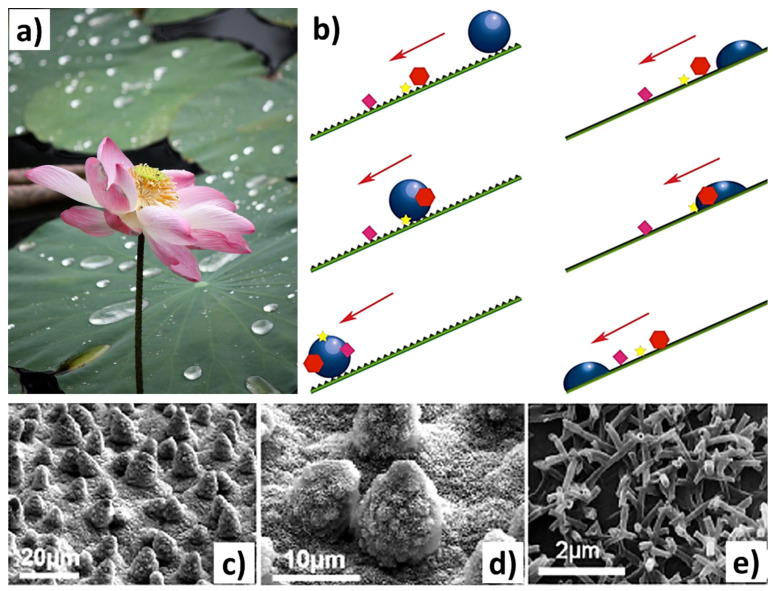
Images showing (**a**) hydrophobicity and self-cleaning characteristics of the lotus leaf surface; (**b**) a schematic representation of droplet movement (red arrow) on an inclined nanostructured superhydrophobic surface, known as the lotus effect. As the droplet rolls off the surface, it collects contaminating particles, effectively cleaning it (left). In contrast, a smooth surface merely redistributes the particles with the moving droplet (right); and (**c**–**e**) SEM micrographs of lotus leaf surface at various magnifications. Reproduced with permission from Ref. [25].

Biomaterial surface modifications, regarding both chemical and topographic approaches inspired by nature, have been explored extensively [43]. Chemical modifications often involve the use of nature-derived compounds, such as biopolymers inspired by mussels, known for their exceptional biocompatibility and adhesive properties [155]. The current section addresses specific surface qualities targeted for reducing bacterial adhesion [88,156], improving cell adhesion, self-cleaning ability [25], including surface roughness, wettability [12], surface energy [147,157], biofouling resistance [113,158,159], and adhesion strength [100,160,161]. Utilizing natural surface topography for modifying these attributes offers advantages as it avoids the release of chemical compounds into the surrounding environment where such surfaces are employed [162]. This topographic alteration in biomaterial surfaces has the potential to reduce the reliance on antibiotics for treating implant-related infections, consequently mitigating the risk of antimicrobial resistance. Figure 9 shows the self-cleaning and hydrophobicity (water-repelling) properties of the lotus surface.

**Figure 10:**
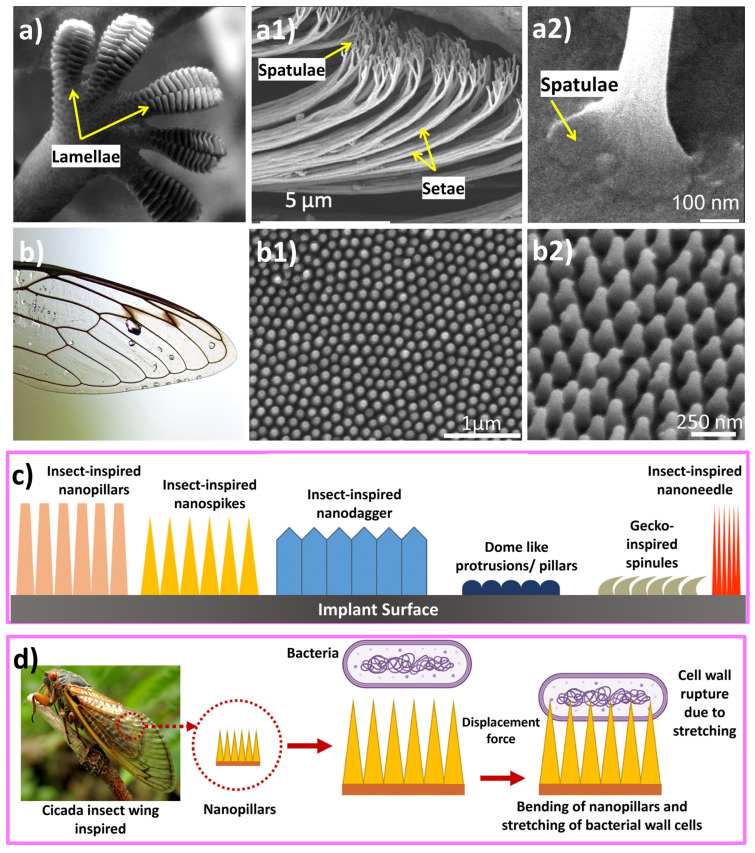
Illustration of (**a**) a gecko foot showing lamellae; (**a1**,**a2**) SEM of setae and hairlike spinules’ structures on gecko toes [170]; (**b**) a cicada wing; (**b1**,**b2**) SEM showing nanopillar-like structure of cicada wing [171]; (**c**) schematic of different types of insect-inspired pillar-like nanotextures used towards fabricating antibacterial surfaces; and (**d**) schematic of bacterial killing/disrupting mechanism on a cicada wing-inspired nanopillar surface [79]. All images were reproduced with permission from respective publishers.

Another aspect is that bacteria can attach to the surface of a material through various mechanisms, resulting in the formation of biofilms when released into a biological environment [113]. To counteract this, various strategies have been devised to disrupt bacterial interactions with surfaces. Topographically textured surfaces offer a chemical-free approach to bacteria inactivation. The effectiveness of bacterial destruction depends on the interactions between bacterial cells and the surface’s texture features (mechanical response), such as pillars, columns, or rods, as shown in Figure 10c. Chopra et al. [79] reported that, when bacteria encounter nanopillars, they undergo a brief downward movement that causes their cell wall to rupture, resulting in lethal damage, as shown in Figure 10d. Bandara et al. [163] suggested a mechanism that involves a combination of strong adhesion forces and shear forces in a mixed short–tall nanopillars topography presence. When bacteria move across taller nanopillars, it causes the nanopillars to bend, which in turn stretches the bacterial cell wall, leading to its rupture. Other attributes like height, width, diameter, and spacing of these features significantly impact the bacterial response upon contact [43,161]. Even more fascinating are the physical alterations that involve creating micropatterns on surfaces to manipulate the hydration layer, rendering them either superhydrophobic or superhydrophilic [102,157,164]. This has given rise to technologies that mimic the texture of shark skin (also observed on lotus leaves) and replicate the superhydrophobic properties, leading to water repulsion and preventing the adhesion of contaminants, contributing to self-cleaning and antifouling properties such as the textures developed on surgical instruments (knife, etc.) [57,113,165,166]. Moreover, superhydrophilic biomaterials, which attract water strongly and facilitate rapid wetting and spreading of liquids across their surfaces, are particularly valued for their ability to promote crucial processes, such as cell adhesion, tissue growth, and fluid transport. Examples of applications include biomedical implants, tissue engineering scaffolds, and microfluidic devices, where the enhancement of these processes is essential for optimal functionality and performance [113,158,167]. Studies have revealed that the interplay between surface topography and various cell types impacts cell morphology, behavior, alignment, migration, and proliferation, among other traits [168,169]. These interactions ultimately alter the dynamics between cells and surfaces. Table 3 lists several bioinspired surface textures fabricated on biomaterials for their added functionality.

**Figure 11:**
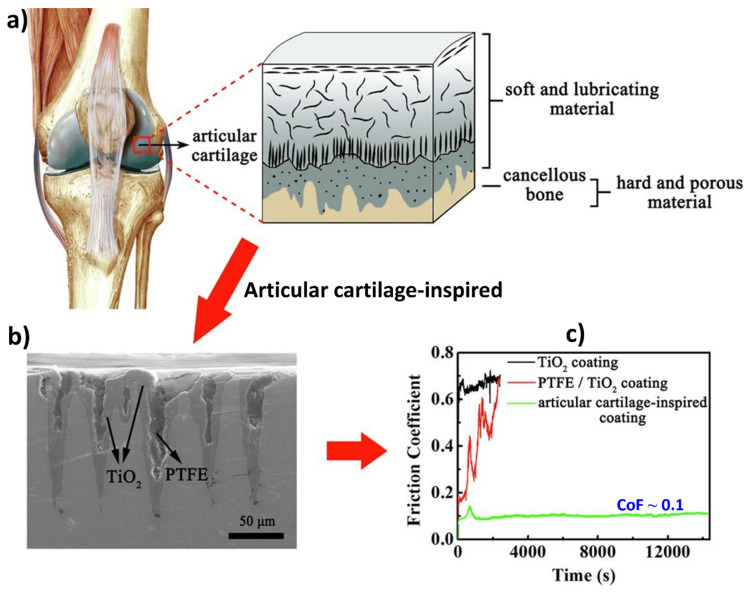
(**a**) Schematic illustration of cartilage organization in joints; (**b**) SEM image of the cross-section of the bioinspired bilayer coating; and (**c**) friction curves of the TiO_2_, PTFE/TiO_2_, and bioinspired bilayer coating under a ball-on-plate reciprocating configuration (counterbody 0.006 m dia. GCr15 steel ball, frequency of 1 Hz, stroke of 0.005 m, and a load of 20 N). Adopted with permission from Ref. [59].

Healthy articular cartilage in joints provides crucial lubrication for smooth movement of human hips or knees [180]. Inadequate lubrication of articular cartilage can lead to degenerative joint diseases, resulting in irreversible degradation of the cartilage. Hence, incorporating stimuli-responsive drug release into lubrication strategies can aid in repairing damaged articular cartilage. In terms of tribological studies, for instance, articular cartilage features a distinctive biphasic structure that incorporates both soft and hard materials to support heavy loads and minimize friction within joints [59,181,182,183,184]. To mimic this structure, Li et al. [59] developed a composite coating consisting of a dual-layer configuration comprising a thin PTFE layer atop a porous TiO_2_ coating on Ti6Al4V alloy, as shown in Figure 11. The top self-lubricating layer reduces friction, while the underlying TiO_2_ layer enhances wear resistance. This bioinspired bilayer coating demonstrates low friction (~0.1), exceptionally high load-bearing capacity (3.2 GPa under nanoindentation of 0.19 N, and a penetration depth of 600 nm), prolonged low-friction durability, and superior wear resistance during dry sliding. These remarkable properties are attributed to a self-repair mechanism, where PTFE is continuously replenished from the pores to the surface by frictional forces, effectively repairing scratches and combating abrasive wear. These findings offer insights into designing intelligent biomimetic coatings with enhanced mechanical properties, thus broadening the scope of applications for biomedical coatings in various fields.

## 5. Bioinspired Surfaces for Erosive Wear Resistance

**Figure 12:**
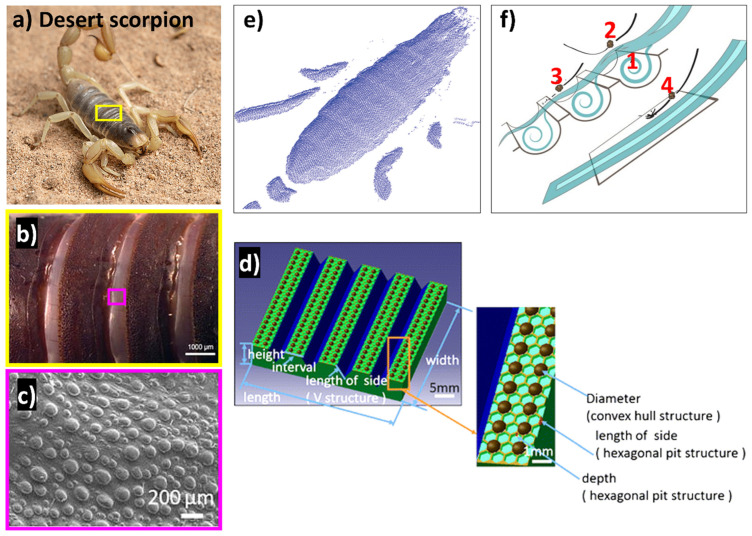
Images showing (**a**) back of an *Androctonus australis* (desert scorpion); (**b**) high-magnification image of desert scorpion back revealing V-groove-like pattern [186]; (**c**) scanning electron microscope image of scorpion back revealing outward bumps on the grooved surface [186]; (**d**) schematic of structure sizes of biomimetic samples; (**e**) point clouds of scorpion back showing bumps on the skin surface, later utilized to model an erosion study [185]; and (**f**) point 1—the swirling air caught in the grooves creates air cushions that slow down the velocity of the wind. This phenomenon reduces the speed of airborne particles, leading to different outcomes; point 2—some particles bounce away without impact, while point 3—others hit the surface at a much slower velocity and with less impact. Point 4—in contrast, a particle hitting a flat surface where the wind velocity remains unaffected will experience a much higher impact [187]. All images are adopted with permission from respective publishers.

Interest in anti-erosion characteristics and mimicked material design inspired by living creatures inhabiting harsh erosive environments, like desert lizards, sandfish, tamarisk, desert scorpions, and dung beetles, has grown gradually [24]. For instance, despite harsh desert conditions where sand particles travel at velocities over 100 km/h, *Androctonus australis*, a north African desert scorpion, remains resilient on the surface, enduring sand-laden harsh winds. Researchers have discovered that desert scorpions resist erosion through their body design. A team from Jilin University in China used 3D laser scanning to create a cloud point representation of the scorpion’s back [185] as shown in Figure 12e. Their analysis revealed that the surface’s convex and grooved design aids in erosion resistance. The grooves cause the air striking the scorpion’s surface to rotate, creating a low-speed reverse flow zone. Essentially, this air flow zone acts like an air cushion, allowing some particles like sand to be harmlessly blown away rather than hitting the surface directly. The particles that do manage to pass through strike the surface with significantly lower velocity and impact angle, reducing the rate of erosion (Figure 12f).

Inspired by the back of the desert scorpion, a few researchers have reported a hexagonal pit structure with excellent anti-erosion properties [186,188,189,190]. The biomimetic samples with microstructures as shown in Figure 12d resulted in reduced erosion wear due to reduced solid particle velocity caused by rotational low velocity (explained in Figure 12f). Similarly, Han et al. [188] explored the erosion resistance of bump and groove shapes inspired by the desert scorpion surface, demonstrating increased erosion resistance compared to smooth structures by approximately 10% and 25%, respectively. Another study by Zhiwu et al. [185] reported that the erosion resistance of the desert scorpion involves a biological coupling approach, combining convex, grooved, and flexible elements. Numerical simulations showed that the grooved surface exhibited superior erosion resistance compared to smooth and convex surfaces, attributed to particle impact velocity, angle, and frequency. Erosion tests confirmed that grooved surfaces performed best at a 30° injection angle. Analysis of groove dimensions revealed that groove distance had the greatest influence on erosion resistance, followed by groove width and height.

Researchers have replicated snakeskin texture features, demonstrating that snake-like skin samples experience significantly less wear than smooth/untextured samples [24,110,124,125,126]. Effectively mitigating erosion wear depends on optimizing the interaction and interference between contributing factors such as geometry, material, and mechanics. For instance, inspired by the head and pronotum surface of the dung beetle, Yang et al. and others [41,97,191] constructed and optimized a dung beetle-inspired textured drill bit surface, achieving better wear reduction and efficiency. However, the exact size and location of the dome and pit structures on the drill bit demand optimization based on soil environment and dung beetle species as the solid particles hitting these structures change trajectory, reducing wear on other structures. However, the relationship between the position and volume of the domes/outside dimples was overlooked and missing.

**Figure 13:**
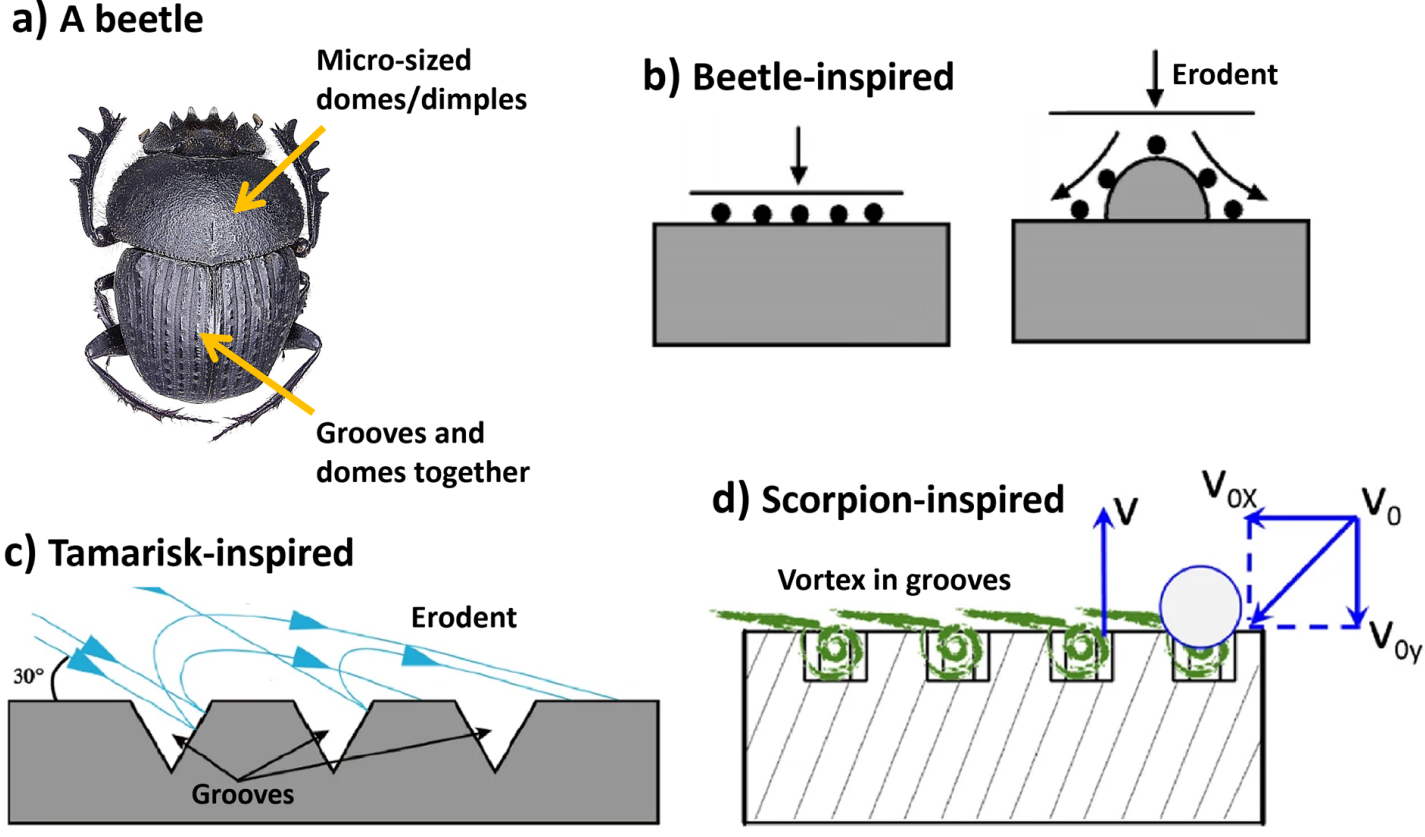
(**a**) Beetle skin surface revealing presence of domes/dimples and grooves [196]; (**b**) schematic diagram of smooth surface and beetle-inspired surface domes in erosion wear [41]; (**c**) schematic diagram of the interaction between tamarisk-inspired surface V-grooves and erodent particles [193]; and (**d**) schematic diagram of the interaction between desert scorpion-inspired grooved surface and erodent particles. Occurrence of rotational flow (vortex) for the impact velocity of particles (Vo), which forms the upward velocity vector perpendicular to the surface (V). The vertical upward velocity V is opposite to the velocity component Voy of the particle in the normal direction, which can slow the normal velocity of the solid particles. Studies show that, even if the rotational flow velocity is small, it still plays a very important role in reducing erosion wear [186]. All images were reproduced with permission.

Tamarisk-inspired V-shaped grooved solid surfaces have been quantitatively studied for erosion rates under particle impact [55,192,193]. These grooves effectively reduce erosion wear within an impingement angle range of 20–60°. However, these impingement angles (global impingement angle) vary due to the ductility of the eroded substance. Jung et al. [194] successfully explained the dependence of the grooved surface’s erosion rate on the impingement angle by considering the diversification of the local impact angle caused by the presence of the grooves. Other factors, such as multiple impacts of particles and air swirls within grooves, have negligible effects on erosion [194]. This suggests both the promise and limitations of this biologically inspired anti-erosive approach. Grooves aggravate erosion when the ambient flow direction is nearly parallel or perpendicular to the surface, which is eroded in a ductile manner. However, one can benefit from the grooves if the surface direction can be adjusted to align with effective anti-erosion impingement angles (i.e., 20–60°). Cylindrical surfaces with grooves exhibit greater erosion resistance compared to smooth surfaces, regardless of wind direction, as observed in tamarisks [30,195]. Yin et al. [193] tested different bioinspired grooves (square, U-shaped, and V-shaped) and found that the V-shaped groove morphology displayed the lowest erosion rate, as shown in Figure 13. However, over time, the bionic structure on the surface degrades, leading to decreased erosion resistance. Other approaches must be explored to limit erosive wear in bionic surfaces as eddy currents on non-smooth surfaces are reduced, eventually leading to erosion resistance loss.

Bionic tribological designs utilize the features of fast-swimming sharks that have skin with scaled structures aligned in the flow direction to minimize friction drag under turbulent conditions [24]. These structures, known as scales or denticles, resemble small dermal teeth and contribute to a 5 to 10% reduction in drag while swimming and are quite widely utilized to develop fluid flow erosive resistance [197]. Luo et al. [48] examined the morphology of shark skin scales and identified factors contributing to frictional drag reduction, including reduced wall friction from microgroove tips protruding into the viscous sublayer, decreased turbulence intensity near the wall due to backflowing microdroplets, a superhydrophobic effect from boundary slipping on the fluid–solid interface, and a nanochain of mucous increasing the thickness of the viscous sublayer to further reduce viscous resistance. Martin et al. [198] studied optimization of riblet geometries inspired by shark skin for minimal frictional drag. Their research emphasized that the reduction in drag was linked to vortices being lifted away from the surface and forming over the riblets, particularly under turbulent flow conditions. This phenomenon contributed to decreased overall shear stress. Fu et al. demonstrated that triangular-shaped riblets offered a more favorable balance between ease of manufacturing and effectiveness in drag reduction [46].

**Figure 14:**
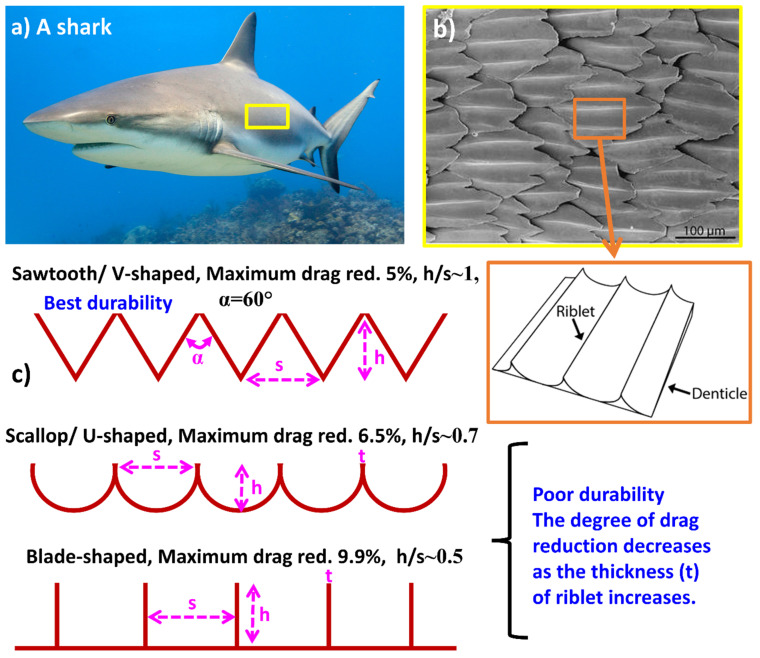
(**a**) Shark skin; (**b**) SEM image of shark skin showing microriblets structure [202,203]; and (**c**) proposed models of shark skin riblets’ structures in the form of a sawtooth (V-shaped groove), scallop (U-shaped groove), and blade (I-shaped groove) [199]. Their optimal structural dimensions leading to improved performance are mentioned as per Ref. [201].

Many studies utilize various models such as V-shaped, U-shaped, I-shaped, scallop-shaped, and blade-shaped structures to replicate the characteristics of shark skin. Airbus applied grooved film to its A320 experimental aircraft, achieving an expected fuel-saving effect of 1% to 2% [199]. Similarly, China’s Y7 aircraft experienced an 8% reduction in drag after being coated with a flow-grooved skin, while applying V-shaped trench film to the surface of the NACA 0012 aircraft resulted in a 6.6% drag reduction [199]. In contrast, the riblet surface exhibited a 9.9% decrease in adhesion resistance compared to the smooth surface [199]. The drag reduction effect was worse as the height of the riblet increased when h > 0.6 μm. Schulz et al. [200] investigated the application of shark skin riblet structures in high-temperature coatings for aerospace blade dynamics optimization. The experimental findings suggested that the zigzag groove spacing (s) should be approximately twice the height (h) of the zigzag groove, with h/s ratios ranging from 0.5 to 0.6 and s values between 15 and 17 μm. These findings offer valuable insights for establishing bionic riblet surface structures for erosive wear resistance. A study by Bechert et al. [201] reported the experimental results on frictional drag-reducing surfaces and their optimization with an adjustable geometry. The results of the study in terms of optimized structural dimensions are shown in Figure 14c.

**Figure 15:**
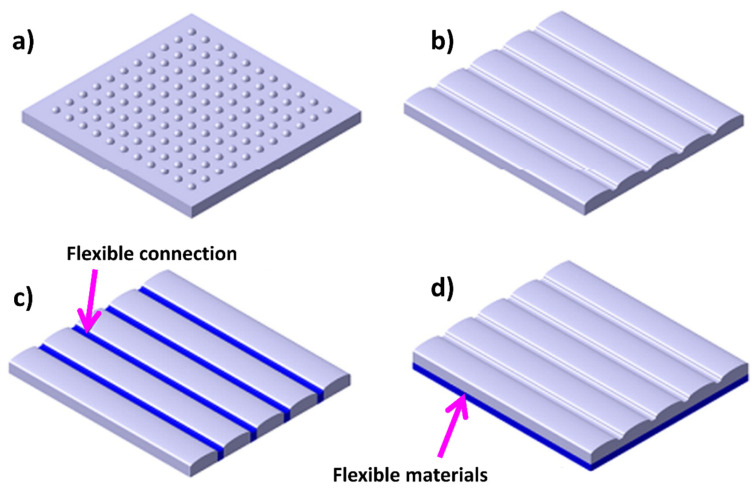
Bionic model of the desert scorpion’s back with (**a**) dome pattern; (**b**) groove pattern; (**c**) coupling bionic models with flexible connection; (**d**) coupling bionic models with a composite structure alternating between soft and hard materials. Reproduced with permission from Ref. [204].

The surface effectiveness of the sharkskin or dorsal of the scorpion in resisting erosion is not solely due to its unique surface morphology but is also influenced by several factors related to coupling function. The protrusions and furrows on the surface serve as morphological coupling elements, while the adaptable linkage acts as a flexible coupling element. The bionic model incorporates these coupling elements, as depicted in Figure 15. By eliminating the adaptable linkage from the model, it was divided into two layers. The upper layer comprises rigid material shaped into a bionic surface morphology, while the lower layer consists of pliable materials. This dual-layered arrangement creates an alternating composite structure of soft and hard materials, which is believed to offer superior erosion resistance, as shown in Figure 15d.

Erosive wear results from solid particles flowing along water or gas, impacting solid surfaces and causing repeated deformations [205]. The complexity of this phenomenon depends on various factors such as the size, shape, hardness, and concentration of erodent particles, as well as the elastic properties, surface hardness, and morphology of the eroded substance, and flow conditions like impacting velocity, angle, and location of impact [51,206,207,208,209]. Investigating erosion wear involves considering erosion rate, penetration depth, load spreading, and stress distribution [24,194]. The lack of a clear understanding of the erosion mechanism hinders the development of a simple, reliable, and universally quantitative erosion model. Despite the growing interest in anti-erosion characteristics and biomimicked material design, the current research in this area remains fragmented. Nevertheless, some significant results are documented in Table 4. Bioinspired surfaces offer erosion wear protection through various mechanisms: enhancing fluid turbulence along the wall, disrupting particle motion, reducing the number of particles impacting the eroded substance, and preventing particle sliding and rolling [194,210,211].

## 6. Bioinspired Structures for Impact or Energy Absorption

**Figure 16:**
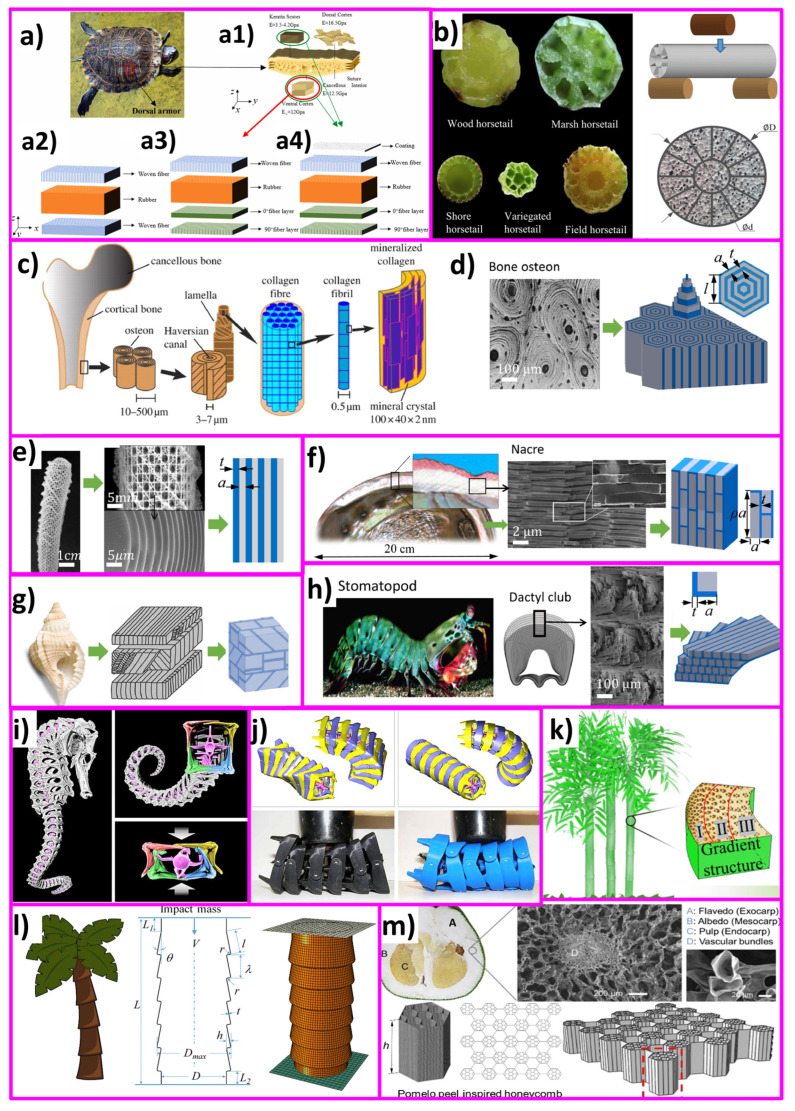
(**a**) The macroscopic morphology of the turtle shell is depicted [49]; (**a1**) illustrates a cross-sectional view of the turtle shell carapace, highlighting its composite layers; (**a2**) ordinary three-layer sandwich structure. Moving to the bionic sandwich structures, (**a3**) fine structure of the ventral cortical layer, resulting in a four-layer sandwich structure, and (**a4**) bionic sandwich structure that mimics the keratin scutes, leading to a five-layer sandwich structure. Reproduced with permission from Ref. [49]; (**b**) horsetail-inspired foamy structures under lateral loading. Reproduced with permission from Ref. [50]; (**c**) bone’s architecture exhibits five levels of hierarchy; (**d**) SEM image illustrates the bone osteon along with a concentric hexagonal unit cell model, (**e**–**h**) optical image, SEM image, and microstructures of sea sponge, nacre, conch shell, and stomatopod dactyl club, respectively. Reproduced with permission from Ref. [213]; (**i**) seahorse skeletons are composed of highly articulated bony plates for flexibility. Reproduced with permission from Ref. [214]; (**j**) DNA-inspired helical structures and their deformations during compression tests. Reproduced with permission from Ref. [215]; (**k**) bamboo-inspired functional gradient structure. Reproduced with permission from Ref. [216]; (**l**) bioinspired conical corrugation tube. Reproduced with permission from Ref. [217]; (**m**) pomelo peel-inspired honeycomb. Reproduced with permission from Ref. [218].

Drawing inspiration from natural organisms such as beetles, woodpeckers, turtles, etc., researchers have explored novel structural designs and materials that exhibit remarkable impacts or energy absorption properties, as shown in Figure 16. By mimicking the unique features and mechanisms found in nature, such as hierarchical microstructures, multilayered compositions, and energy dissipation mechanisms, bioinspired structures offer promising solutions for mitigating impact forces, enhancing structural resilience, and sometimes improved flexibility. 

The turtle shell abaxial armor, comprising lightweight and robust natural composite laminated material, typically consists of three to six layers. Its macro-mechanical properties are influenced not only by its multilayer structural characteristics but also by its intricate multiscale and multilayered microstructure and layering methods. The complex morphology of the turtle shell has been extensively studied by several researchers [49,219,220,221,222,223,224,225] to understand its superior protective capabilities against environmental penetration. Pangolin shells offer similar characteristics to turtle shells and have been studied by several researchers [24,135,149,226].

Beetles’ elytra serve as lightweight yet rigid structures, effectively shielding beetles from external impact loads, capable of withstanding an impact velocity of up to 1 mm/min [227,228]. The superior mechanical properties of the elytra are attributed to the irregular cellular pattern observed in the laminated cross-section, featuring hollow pillars at the wall and intersections. This pattern includes circular cross-section fibers sparsely distributed around the outer edge, transitioning to rectangular structures with a denser distribution towards the middle lamination. Furthermore, the beetle’s forewing is reinforced by trabecular structures between different laminas, significantly enhancing inter-laminar strength, approximately 30 times higher than that of pure chitin fiber laminas [228]. Some studies on beetle inspiration have shown its tremendous benefits regarding the impact-absorbing structure [41,70,97,227,229,230].

Woodpeckers demonstrate remarkable shock absorption capabilities when they drum rhythmically with their beaks. They can peck at a frequency of 18–22 times per second and around 12,000 times a day on average, with each peck lasting approximately 50 ms. Despite the high impact deceleration and a repeated impact velocity of 6–7 m/s during the pecking process, no head injuries are observed afterward [228,231]. Ha et al. [231] created a woodpeckers’ beak-inspired novel wavy honeycomb sandwich panel and investigated its structural design approach by adjusting wave number and amplitude. This innovative design outperformed the traditional hexagonal honeycomb sandwich panel in energy absorption performance.

The mantis shrimp, known for its powerful raptorial projections, utilizes its sharp dactyl club as a biological hammer for smashing prey and defending against predators. With extreme accelerations facilitated by a unique power amplification mechanism, it can strike both hard-shelled and soft-bodied prey with remarkable force. Particularly, the peacock mantis shrimp can achieve accelerations of over 105 m/s^2^ and impact velocity exceeding 20 m/s, making it one of nature’s most formidable and fastest impact scenarios [228]. Most of the notable studies on shrimp-inspired energy-absorbing structures include [114,228,232,233,234,235].

Inspirations from various fruits and plants, such as nutshell, pomelo skin, bamboo structure, and horsetail, present promising opportunities for designing impact-absorbing structures. The pericarp, or fruit wall, of all angiosperms consists of three layers: the outermost exocarp, followed by the mesocarp, and the innermost endocarp. In nuts, the pericarp dehydrates entirely, resulting in a highly lignified, mechanically robust compact shell. On the other hand, in drupes, only the endocarp undergoes lignification, forming a resilient protective layer around the seed.

Yang et al. [236] studied pomelo peel and its bionic PEEK structures, finding that different varieties follow similar porosity trends: lowest near the endocarp and exocarp, highest in the mesocarp. The location of maximum porosity varies by pomelo variety. During quasistatic compression, deformation initiates in the highest porosity region, with a near-zero Poisson’s ratio, while the region near the exocarp remains less compressed with a positive Poisson’s ratio. Additively manufactured porous PEEK cubes and numerical simulations confirm the benefits of porous design from pomelo peels for efficient energy absorption, offering clear guidance for designing lightweight materials with high energy absorption.

Chen et al. [237] investigated three bioinspired hierarchical self-similar structures based on basic shapes (curve, circle, and hexagon). They assessed energy absorption rates of unit cells with varying structure ratios through experiments and simulations. Honeycomb and bamboo fractal structures outperformed snake-like fractals in energy absorption. The bamboo fractal structure displayed the highest energy absorption capacity and was used to construct carbon fiber-reinforced composite structures. The numerical findings showed that a seven-cell bamboo fractal structure effectively represents the energy absorption ability of a self-similar structure with an array of cells. Overall, the 3D-printed self-similar composite sandwich structure demonstrates significant potential as a lightweight energy absorption structure.

In terms of aircraft wing’s navigation where energy absorption is as important as other mechanical parameters such as flexure strength, etc., Prakash et al. [238] developed a cashew nut-inspired bionic structure using 3D-printed high-stiffness lignin–ABS core and industrial hemp with aluminized glass fiber epoxy skin. The addition of 30 vol% Al-glass and hemp fiber with lignin-strengthened ABS core (60 vol%) significantly enhanced the mechanical properties of the composite. This composite demonstrated impressive tensile strength, flexural strength, Izod impact resistance, interlaminar shear strength, and compression strength. Table 5 records several bioinspired structures reported in recent literature for their superior impact or energy-absorbing capabilities.

A few promising examples of such inspiration applied to material structuring are described below and systematically compiled in Table 5.

Several features combine to influence the impact resistance of a material and its response to dynamic loading: hierarchical structure (discrete structural elements across multiple length scales); composition (distinct interfaces between a stiff phase for rigidity and strength and a softer phase for ductility); and porosity (gaps filled with air or fluid across all length scales).

Additionally, biological materials demonstrate viscoelastic and/or viscoplastic behaviors, characterized by time-dependent stress and strain responses and time-dependent permanent deformations, respectively [251]. These behaviors are attributed to the polymeric constituents present in biological materials, such as collagen, keratin, cellulose, hemi-cellulose, lignin, and chitin, which determine their ability to absorb and dissipate energy under dynamic conditions. These characteristics are ubiquitous across biological materials. Viscoelastic materials exhibit both elastic and viscous responses, where energy is stored during deformation and dissipated as heat upon loading, respectively. This time-dependent behavior allows viscoelastic materials to attenuate shock and dampen vibration, contributing to impact isolation. For instance, the viscoelastic response of muscle and tissues surrounding the woodpecker’s hyoid apparatus reduces stress waves induced by pecking, while articular cartilage serves as a viscoelastic barrier against high-speed loading, limiting potential damage to surrounding tissues [252,253]. Despite the inherent viscoelasticity of all biological materials, alternative energy absorption mechanisms may prevail at high strain rates, influenced by structural factors such as layering and void distribution, as observed in wood’s impact resistance mechanisms [231].

Furthermore, many biological systems exhibit specific impact-resistant structural elements predominantly on the micro- and mesoscale, including layered, gradient, tubular, sandwich, and sutured structures [251]. These elements, often found in combination, contribute to remarkable properties under dynamic loading conditions. While serving as foundational components for hierarchical impact-resistant structures, individual tests using computer simulations, 3D printing, and composite prepregs have shown that each of these arrangements can enhance a material’s impact resistance independently [217,229,233,249,254].

**Figure 17:**
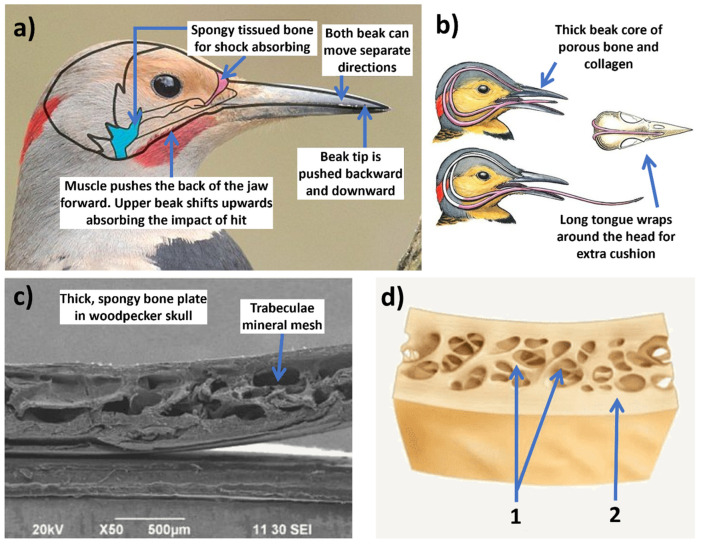
Images showing (**a**) a woodpecker’s head and beak; (**b**) tongue wrapping around the brain to provide extra protection from impact; (**c**) SEM image of spongy bone situated in between brain and beak that absorbs impact (pink area in part (**a**)); and (**d**) schematic of bone showing 1—spongy bone; 2—compact bone. Images adopted CC BY-NC from Science China Press and asknature.org (Accessed on 12 February 2024) [255].

A very good example is the woodpecker, as shown in Figure 17. When the woodpecker strikes an object with its beak, the immense force at the tip is cushioned by its beak structure and the resilient hyoid bone [255,256]. Consequently, the impact’s stress is significantly diminished from the beak’s tip to where it connects with the skull, reducing the force that reaches the bird’s cranium. The cranial bone, consisting of dense compact bone enveloping layered plate-like structures, forms an efficient shock absorption system [257]. When encountering forces, this deeper bone disperses frequencies in various directions away from the central impact point. Although fragile on its own, the deeper bone, encased in compact bone, maintains flexibility within, absorbing shock during movement. Furthermore, the woodpecker’s brain, proportionally small with a high surface area to weight ratio, further disperses impact force over a larger area, minimizing damage. Computer simulations suggest that only a minute fraction of the impact ultimately reaches the brain, bolstered by the bird’s lengthy tongue, which envelops the brain for added protection [253].

## 7. Bioinspired Design of Tools for Cutting or Machining

**Figure 18:**
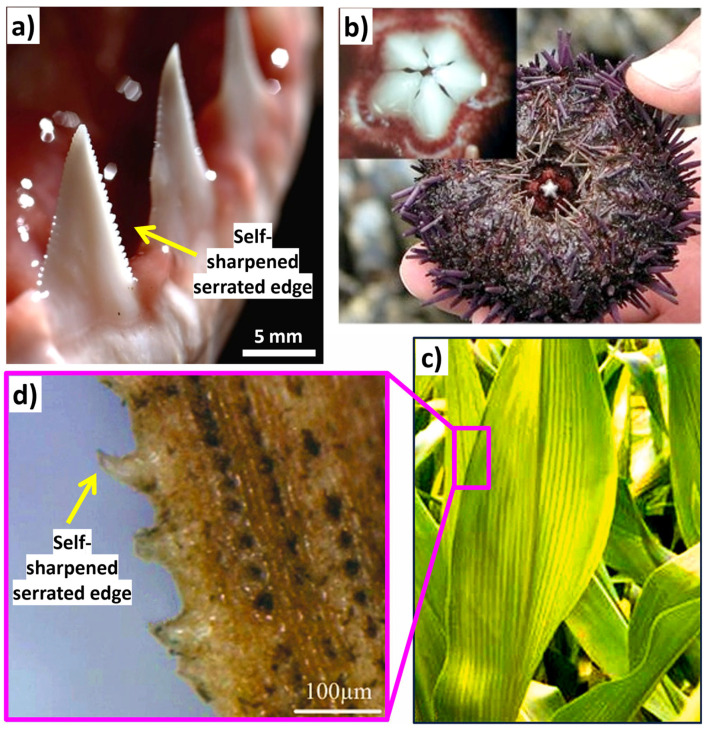
Images showing self-sharpened serrated edges in (**a**) shark teeth [290]; (**b**) rock-eating sea urchin’s teeth [291]; and (**c**,**d**) corn leaf [289]. Images were reproduced with permission.

Bionic enhancements of cutting tools (soil, rock, metal, and biological) enhance tool durability, reduce friction, increase heat dissipation, and improve material/surgical removal rates. For example, soil adherence on soil excavation equipment poses a significant technical challenge due to soil characteristics. Metal cutting tools endure significant mechanical and thermal stresses due to the hardness and strength of metals, leading to considerable abrasion and chip adhesion [258]. This wear ultimately diminishes the tools’ lifespan and compromises the quality of the workpiece during processing. This phenomenon elevates traction force by over 30% and concurrently diminishes productivity by the same margin [259]. In such cases, biomimetic designs inspired by the structure of animal teeth or plant leaves have been explored to increase wear resistance and reduce energy consumption during drilling operations. In rock-cutting tools or tunnel-boring machine applications, bioinspired surface modifications based on the properties of animal skins or plant surfaces have been investigated to reduce friction between the cutting tool and the tunnel surface, leading to greater mechanical traction, smoother excavation, and reduced tool wear. Moreover, features like anti-sticking properties for drilling the soft coal or earth [41,260], reducing sticking of drilling-breaks [41,261,262,263,264,265], reducing soil wear and adherence [41,259,266,267,268], self-cleaning [25,73,204,269,270], self-sharpening [271,272,273], self-lubricating [76,138,142,144], self-cooling [274], erosion protection (dry and slurry) [24,211,275,276], increased flexibility [24,277], reducing tool-chip friction [278], decreasing contact length between tool and chip [263,278], changing chip flow direction, improving adhesion resistance [88,266], stabilizing the built-up edge, lowering adhesive wear between tool and chip [105], etc., are shown to be attainable with specific biomimetic surface designs on machining/cutting tools. Additionally, some studies have suggested that a synergistic approach of solid lubricant and the bioinspired textures on tools (turning, etc.) can greatly enhance their performance due to considerable reduction in turning/milling/drilling force and temperature, resulting in major reduction in tool wear [138,270,279]. Some existing examples and nature inspirations reported are derived from python skin [123,274], sea urchin teeth [44,272,273], crab or dung beetle claw and legs [21,41,228,280], mole pelt claw [24,36,268,281,282], pangolin claw [24,52,226,277], wood wasp ovipositors [265,280], earthworm skin [262], seashell [283,284], human teeth [285,286], sunflower seeds [259], corn/maize leaf [287,288,289], badger teeth [36,44,271,273,285], etc. Figure 18 shows the self-sharpened serrated edges in different biological species.

**Figure 19:**
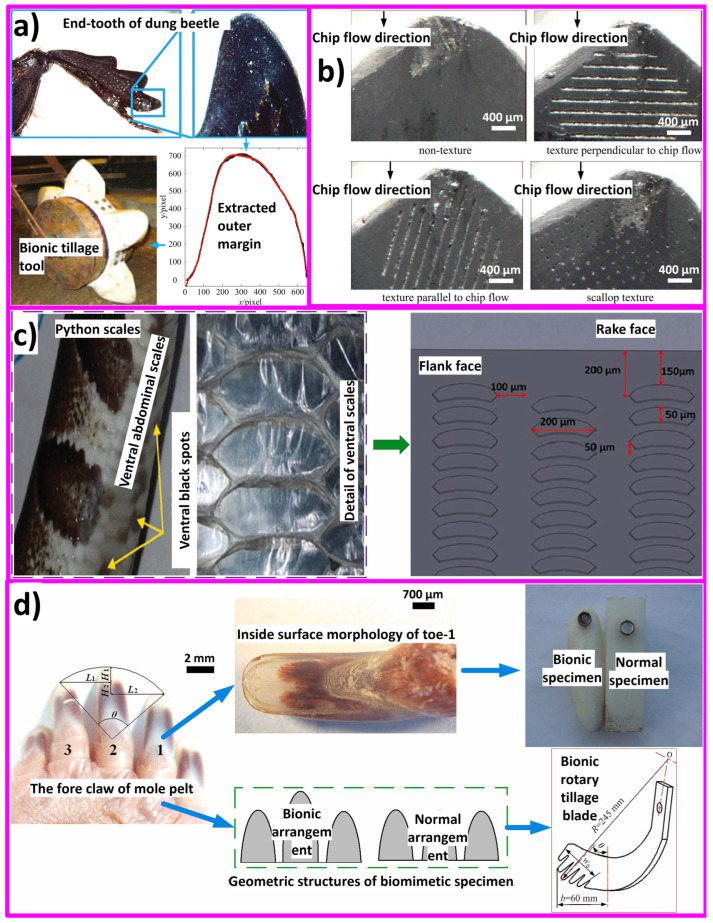
(**a**) Dung beetle-inspired design of tillage toothed wheel [35]; (**b**) various bionic texturing on rake face of a turning tool [259,293]; (**c**) snakeskin-inspired bionic turning tool [294]; and (**d**) bionic soil cutting tools inspired by claw toes of mole pelt [282]. All images were reproduced with permission.

In agricultural and other machining or cutting tools, a certain level of cutting resistance is expected and necessary for the tool to effectively perform its task. However, excessively high cutting resistance and soil adhesion can lead to increased working resistance, increased wear on the tool, and reduced cutting efficiency, significantly reducing service life [292]. Certain soil-burrowing animals, notably dung beetles, ants, and mole pelts, thrive in moist or sticky soil environments yet manage to keep their shells clean and free from soil adhesion. They achieve this anti-adhesive property through the evolution of unique non-smooth surfaces. Moreover, due to their claw’s low soil-cutting resistance (a measure of the difficulty or effort required for the tool to move through the soil medium) and soil adhesion, they can easily dig long tunnels within a short period of time. Hence, the foreleg of the dung beetle and mole pelts serves as an effective bionic prototype for soil-cutting tools as well. For instance, Zhang et al. [35] fabricated a toothed gear inspired by the intricate outer contour curve of the foreleg end-tooth of a dung beetle (Figure 19a). The bionic toothed wheel significantly diminishes draft force by up to 16.5%. Finite element analysis demonstrated that the apex of the bionic peak tooth experiences the highest stress concentration at the contact point with the soil, leading to enhanced cutting tool performance and reduced adhesion and friction between the tool and soil material [35]. Ji et al. [282] created a bionic soil cutting model with ABS polymer, mimicking the foreclaw toes of a mole pelt through reverse engineering and 3D printing. The bionic model exhibited a 12.8% reduction in cutting resistance compared to a standard specimen.

In machine tools including turning, drilling, milling, etc., the bionic texture generation on the tool’s surface enhances its tribological characteristics, including friction, lubrication, and wear, at the interfaces with the workpiece. This texture not only decreases friction by reducing the contact area between the chip and the tool interfaces but also entraps wear debris particles during cutting operations, reducing the plowing action of the tool, and further decreasing friction between the interfaces. Several other benefits, such as lubricant storage in the textures, etc., have already been discussed in the above sections. For instance, the rat, a prevalent rodent known for its strong incisive teeth, possesses remarkable metal cutting/machining abilities. Researchers discovered that the “rake face” of the rat’s incisors featured a unique “crescent depression” morphology. This morphology, far from compromising structural integrity, enhanced the sharpness of the cutting edge, thereby bolstering cutting/machining efficiency. Drawing inspiration from this, it was shown through turning simulations and experiments that these bionic-gradient microtextured turning tools effectively reduce the primary cutting force while simultaneously enhancing structural resilience [295,296]. Another widely reported and effective inspiration comes from sea urchin teeth offering insights into self-sharpening mechanisms due to their ability to remain sharp when eating stone [272]. Three noteworthy aspects of these teeth are the following: (i) despite being composed of calcite, they can grind limestone, which shares a similar composition; (ii) the three structural elements of the teeth—plates, fibers, and the polycrystalline matrix between them—exhibit highly aligned orientation from the nanometer to the centimeter scale; and (iii) the sea urchin teeth exhibit self-sharpening properties with use, rather than dulling [273]. It was revealed that the tooth’s structure determines the location of fracturing and renewal, resulting in self-sharpening. Fracturing is initiated by compressive or shear stresses, with cracks propagating through the plate, shedding off a portion along with its adjacent polycrystalline matrix [271]. In another inspiration taken from dung beetle skin, Siju et al. [297] employed laser biotexturing to create microgrooves, microdimples, square texture, and a combination of both on the carbide tool’s rake face. These textured tools were then used for turning operations on Ti-6Al-4V alloy. The study found that the dual dimple and grooved textured tool exhibited superior performance in reducing friction, shear angle, and the chip reduction coefficient. Li et al. [298] replicated shark skin textures on the tool’s rake face and nanotextures on the flank face of a carbide tool with WS_2_/C coating. They observed that the shark-textured tool, parallel to the cutting edge, exhibited reduced flank wear, temperature, and surface roughness. Fatima et al. [294] imitated hexagonal textures from ball python skin on the tool’s flank face during turning of AISI 4140 steel. They found that the bioinspired textured tool outperformed in reducing contact length, temperature, and cutting forces. Liu et al. [299] created microgrooves in parallel, perpendicular, and inclined orientations on the carbide tool’s flank face, inspired from dung desert scorpion. They investigated the impact of these textured tools during the turning of green alumina ceramics and found that the parallel grooved tool displayed reduced flank wear compared to the conventional tool.

**Figure 20:**
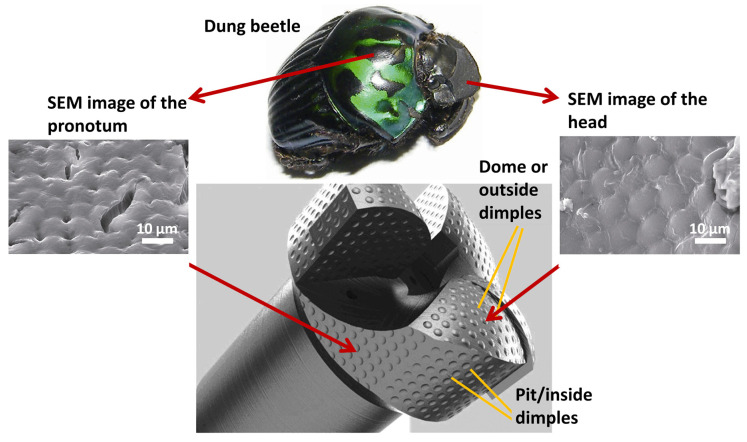
Bionic drill bit inspired by dung beetle. Reproduced with permission from Ref. [41].

**Figure 21:**
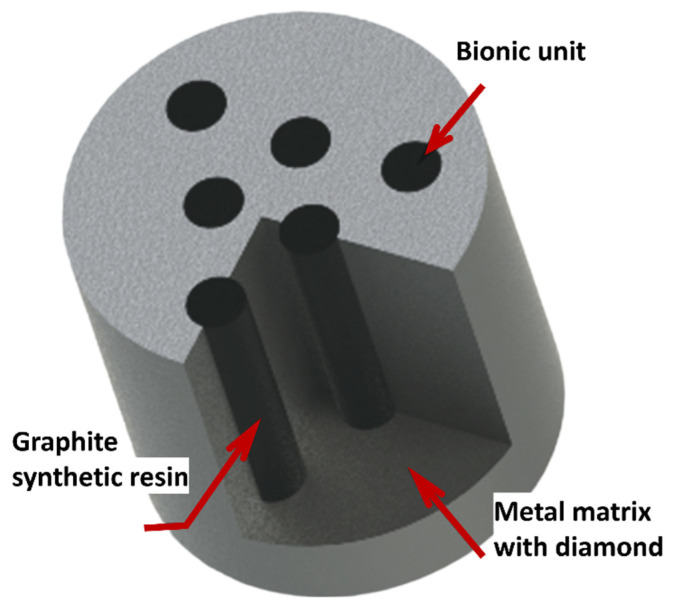
Bionic impregnated diamond bit inspired by dung beetle and earthworm to enhance lubrication mechanism. Reproduced with permission from Ref. [191].

In earth drilling/tunneling works, a coal seam, being a soft rock layer, poses challenges in drilling due to hole collapses and the tendency of rock powder to stick to drill bits [300]. To address these issues, Yang et al. [41] developed a cemented carbide drill bit inspired by the dung beetle as shown in Figure 20. The domes on the bit minimize adhesion, while the pit accommodates detritus. Moreover, the curves facilitate detritus removal from the cutting area. With this bionic drill bit, the drilling speed rose by 45%, and the average abrasion rate dropped by 23%. Gao et al. [300] developed a bionic bit inspired by a pangolin claw to enhance cutting performance in drilling hot dry rocks with high hardness and poor drillability. Experimental drilling of hot dry rocks demonstrated around a 97% improvement in drilling rate and almost 1.2 times longer lifespan compared to the non-bionic bit (the statistical deviations in the reported results were missing). Similarly, Wang et al. [264] utilized the pattern from mole claws to design a polycrystalline diamond compact (PDC) rock cutter with a multistep microarc-structured diamond edge. This bionic PDC rock cutter exhibited remarkable anti-abrasion performance, increasing the service life by over three times and improving the drilling efficiency by approximately 200%. Going an extra mile, Wang et al. [191] introduced a novel bionic bit (Figure 21) inspired by the morphological traits of a dung beetle, featuring concave structures, and the abrasive wear (resistance) and other functional attributes of an earthworm, such as for a continuous lubricated surface texture. The bit was basically a composite of epoxy, graphite (70 wt.%), and diamond as reinforcement. The synergistic multitextured bionic bit was reported to offer high wear resistance and improved cutting capability (due to the continuous exposure of diamonds in the composite after epoxy worn out) in comparison to those with common surface. The graphite (solid lubricant and soft non-metal) composing the bionic unit possesses the ability to self-regenerate the concaves, providing a trapping effect for rock particles as shown in Figure 21. Additionally, the bionic surface enlarged the surface area, leading to enhanced heat dissipation and increasing the frictional contact interface.

**Figure 22:**
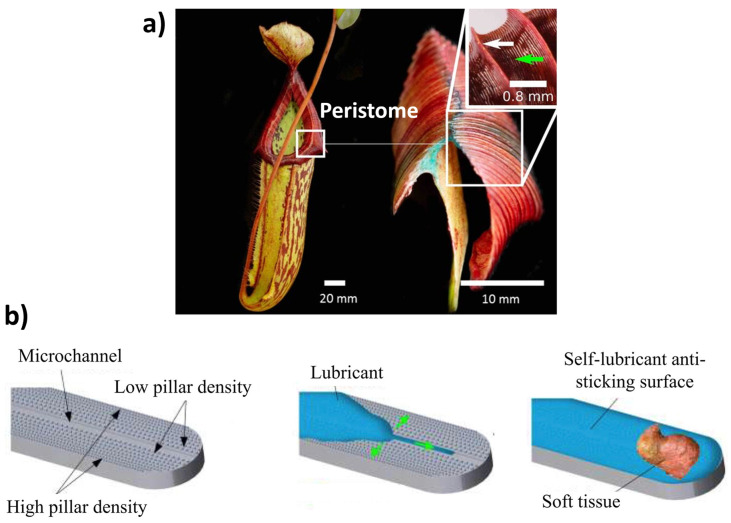
(**a**) A portion of the Nepenthes peristome showing both the inward (left) and outward (right) slanting regions and their microscopic grooves; and **(b)** self-lubricating mechanism of bionic design of electrosurgical knife inspired by Nepenthes [60,106,302].

In the biomedical field, surface modification of medical devices, particularly electrosurgical scalpels, has garnered considerable interest for its simplicity and minimal tissue trauma [106]. Introducing bionic texture to the tool’s surface enhances hydrophobicity, increasing the contact angle and aiding faster fluid flow. This results in reduced temperature and adhesion during the surgical cutting process. Particularly, in orthopedic surgery, medical drill bits play a crucial role in pre-drilling. Incorporating bionic textures on these tools can effectively reduce drilling forces, optimize stress distribution, and mitigate stress concentration issues, ultimately enhancing the quality of pre-drilling procedures [263]. A promising method involves integrating harmless solid lubricants into the bionic texture of medical tools to improve cutting execution and ease patient pain [42]. For instance, Liu et al. [60] explored bionic designs inspired by the hydrophilic microstructure of Nepenthes openings to develop self-lubricating electrosurgical scalpels. By electrolytic etching, a wetting gradient was achieved on the scalpel’s surface, featuring non-uniformly distributed cylindrical patterns/textures. This design facilitates rapid lubricant diffusion and coverage, effectively reducing adhesion. When used for biological surgeries, the surface texture of the bionic tool could retain a significant quantity of lubricant. This transformation changes the initial solid/solid contact to solid/liquid/solid contact, effectively reducing tissue adhesion by approximately 80%, tissue adhesion volume by about 88%, wound damage area by around 81%, and thermal damage area by about 70% [60,270,301]. Moreover, the slippery zone of Nepenthes has many crescentic arc structures, which have been shown to have drag reduction and hydrophobicity [269]. Figure 22 shows the Nepenthes-inspired design of an electrosurgical knife and its features.

Table 6 summarizes recent results from the literature concerning various bioinspired designs exploited for the enhancement of cutting or machining tools.

## 8. Other Tribological Applications

### 8.1. Biomedical

Incorporating bioinspired textures like microgrooves or nanopillars on drug delivery implants or devices can enhance their tribological properties, improving performance and reducing tissue damage during insertion and operation [308,309,310]. These textures also facilitate controlled drug release by enhancing interaction with biological tissues, leading to more efficient drug delivery and better therapeutic outcomes [311]. Another application is in the design of multifunctional medical textiles for drug therapy [312], where optimized textile structures (dermatological) and material properties are crucial for achieving desired frictional conditions between human skin and textiles. This optimization includes considerations such as pressure relief, friction, water transport, and encapsulation of drug molecules like cyclodextrins or ibuprofen for controlled release to the skin over time [313,314,315,316]. Apart from drug delivery [31,82,85], dermatological applications [317], and implants [318], contact lenses, for example, rely on tribological principles to ensure comfort and proper function. The interaction between the contact lens material and the surface of the eye must be carefully engineered to minimize friction and ensure smooth movement of the lens over the cornea. This involves considerations such as surface wettability, lubrication, and biocompatibility to prevent irritation and damage to the delicate tissues of the eye [319,320,321,322,323,324].

### 8.2. Soft Robotics

Taking inspiration from various animals, such as reptiles, caterpillars, earthworms, inchworms, and starfish, researchers have explored the use of highly deformable and soft materials in robotics. These materials enable robots to perform a wide range of movements, including crawling, swimming, flying, and legged locomotion, while also allowing for functions like controlled adhesion (grip), mechanical anisotropy, gas exchange, thermal control, etc. [325]. Unlike traditional rigid and metal-based robots, soft technologies offer adaptive and flexible interactions with unpredictable or dynamic environments. Particularly in challenging terrain and spaces, mobile robots benefit greatly from these characteristics [20,24]. When it comes to gripping objects, soft grippers rely on compressive force and friction, with the surface topography playing a significant role.

Inspired by the stretchable nature of snakeskin, researchers like Lamping et al. [326] have investigated the frictional properties of snake scales and the softness of the underlying material. They found that the soft base material increases friction anisotropy and reduces inconsistencies in hand manufacturing. In another study, Hao et al. [327] explored the concept of fingerprint-inspired surface textures, such as whorls, loops, and arches, to enhance the performance of soft robotic hands in lubricated conditions. They found that applying fingerprint-like films to the fingers of soft robotic hands improved their gripping performance in lubricated conditions. This research highlights the effectiveness of surface texture design in regulating the grasping capability of humanoid robotic hands. Few other studies based on frictional behavior (surface adhesion/locomotion) of bioinspired robots exist that describe tactile perception and other functionalities, including tunable mechanical/tribological response [328], sensitivity to changes in external stimuli (frictional feedback for improved maintenance, etc.) [329], biotribology [326], self-healing mechanisms [330], etc.

Use of biomicro/nanorobots for biomedical applications, including soft tools for surgery, diagnosis and drug delivery, wearable and assistive devices, prostheses, artificial organs, and tissue-mimicking active simulators for training and biomechanical studies, are vital [331,332,333]. For instance, in cases where traditional surgical tools are impractical, such as in deep brain, liver, or organ therapy, soft robotics offer a promising solution [333]. Bioinspired surface-textured robots can be guided to specific locations and release drugs using external stimuli like magnetic fields or ultrasound [333]. This targeted drug delivery enhances therapy effectiveness in hard-to-reach areas.

### 8.3. Space

**Figure 23:**
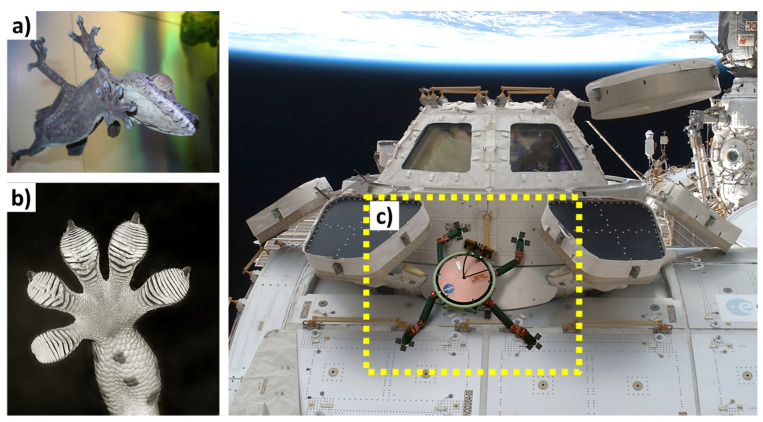
Images showing (**a**) a gecko; (**b**) microscopic gecko’s toepads with millions of microscopic hairs [170]; and (**c**) Limbed Excursion Mechanical Utility Robot (LEMUR, within a highlighted yellow border.) climbing around the outside of the International Space Station. JPL considered outfitting LEMUR with its gecko-imitating gripper technology. Images reproduced from NASA [338].

In the field of space exploration, bioinspired tribology plays a pivotal role in ensuring the dependable performance of mechanical components and systems amidst extreme conditions like vacuum, microgravity, temperature fluctuations, lubricant evaporation, and exposure to cosmic radiation. For example, surfaces modeled after the micro- and nanostructures found on shark skin exhibit decreased friction drag and improved hydrodynamics, offering valuable insights for crafting spacecraft surfaces and components that minimize aerodynamic resistance during launch and re-entry maneuvers. By drawing inspiration from the feet of certain animals or the treads of insects, scientists can devise structures and surface textures for space rover wheels that optimize traction, minimize slippage, and withstand wear and tear while traversing the challenging terrains of extraterrestrial landscapes. For instance, replicating the specialized gripping mechanisms observed in gecko feet, which utilize microscopic hairs to adhere to surfaces, could enhance a rover’s capability to navigate steep slopes or uneven ground on celestial bodies such as Mars or the Moon [334]. Furthermore, bioinspired surface coatings mimicking the self-cleaning attributes of lotus leaves could deter the accumulation of dust and debris on rover wheels or spacecraft bodies, ensuring optimal traction and minimizing frictional heating while reducing the likelihood of mechanical malfunctions. Notably, the combination of shape memory alloys and bioinspired surfaces presents synergistic prospects for developing advanced tribological solutions with adaptable microstructures and functionalities, allowing for customized friction and wear properties in response to the fluctuating environmental conditions encountered in space [335,336,337]. Figure 23 shows LEMUR with its gecko-imitating gripper technology climbing around (yellow dashed area).

### 8.4. Renewable Energy

Bionic engineered materials/surfaces play a crucial role in advancing the transition to a net-zero economy by offering breakthroughs in various renewable energy sectors, including superior energy efficiency and sustainability through minimizing friction and wear while enhancing the energy produced. For instance, erosion wear of solar cells requires focus, especially in desert lands, and space where the phenomena can be catastrophic and lead to complete equipment failure [339]. In wind turbines, the wind blades often undergo erosion/impact through solid-particle or slurry processes or hurricanes (when installed in sea) [340]. In offshore wave energy converters, bearing failure by wear is a common issue resulting in downtime of the whole system. To this end, bionic enhancement of solar cell, wind turbine, or hydro bearing surface has been shown to facilitate more efficient capture of solar energy, enable the development of superior wind turbine blades, and enhance the energy efficiency of tribological generators, etc.

**Figure 24:**
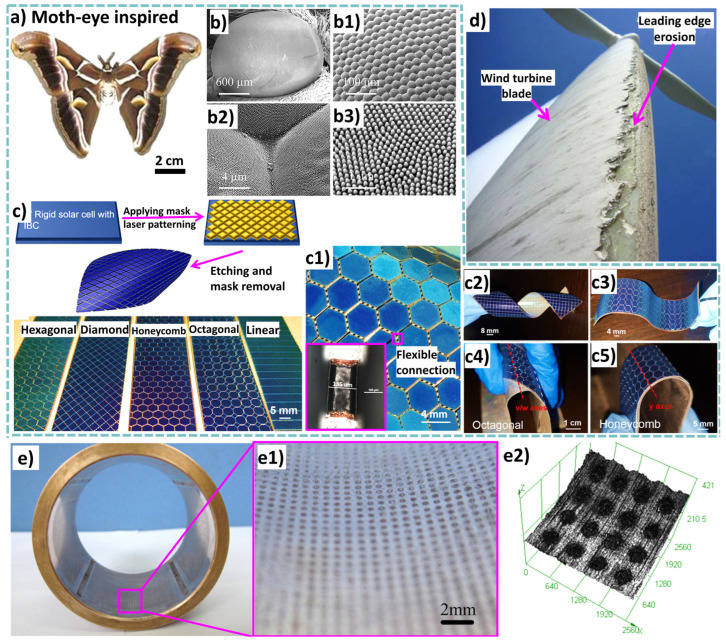
Images showing (**a**) a moth [341]; (**b**,**b1**,**b2**,**b3**) SEM images of moth eye at different magnifications [341]; (**c**) preparation step and optical image showing the five corrugation patterns that are studied, including hexagonal, diamond, honeycomb, octagonal, and linear structures [47]; (**c1**) optical and SEM images of the hexagonal corrugated solar cells for enhanced flexibility [47]; (**c2**,**c3**,**c4**,**c5**) optical images of the diamond, hexagonal, and honeycomb corrugated photovoltaic cells flexed in different axes [47]; (**d**) leading edge erosion in a wind turbine blade [342]; (**e**) a bearing [343]; (**e1**) enlarged photo of the texture on the inner surface of the bearing [343]; (**e2**) surface morphology of the texture as illustrated by laser scanning confocal microscopy [343]. All images were reproduced with permission from their respective publishers.

Especially in the area of solar cells, accumulation of dust (soiling) and repetitive impact of sand particles (erosion) affect the performance of solar power technologies. Replicating natural structures such as those found on lotus leaves or moth eyes can lead to enhanced light absorption, self-cleaning capabilities, hydrophobicity, and reduced reflection on solar panels while contributing to lower erosion rates [45,339,344,345,346], as shown in Figure 24a,b. By minimizing surface reflection, improving light trapping, and mitigating erosive wear, these textures ultimately enhance the efficiency and longevity of solar cells, resulting in higher energy yields. Furthermore, Atab et al. [47] showcased an innovative approach for creating ultraflexible, lightweight, and highly efficient (energy conversion) monocrystalline silicon solar cells. They achieved excellent reliability, mechanical resilience, and thermal performance by employing a corrugation method in conjunction with moth eye-inspired bionic laser patterning, as shown in Figure 24c.

Leading edge erosion (Figure 24d) is a significant issue for wind turbine blades, often caused by factors like insect impacts, dust accumulation, or ice formation [56,347,348]. These factors create surface roughness, affecting the aerodynamic performance and leading to energy production losses. Maintenance costs also increase as a result of this erosion [38]. To address this challenge, researchers have explored the potential of bionic textures on wind turbine surfaces to enhance longevity and energy efficiency. For example, Lv et al. [45] demonstrated that bionic-designed textures inspired by natural surfaces like lotus leaves, water strider legs, or owl feathers can trap air effectively [349], reducing the interaction with liquid water and preventing erosion from water droplets or ice adhesion (anti-icing functionality). These textures also offer additional benefits, such as self-cleaning capabilities, inspired by features like the lotus leaf, which help to keep dust off the blades and maintain aerodynamics, as well as noise reduction inspired by owl feathers [340]. Moreover, mimicking surface topographies from natural structures like shark skin or penguin skin can minimize drag and increase lift, resulting in significant improvements in energy output.

**Figure 25:**
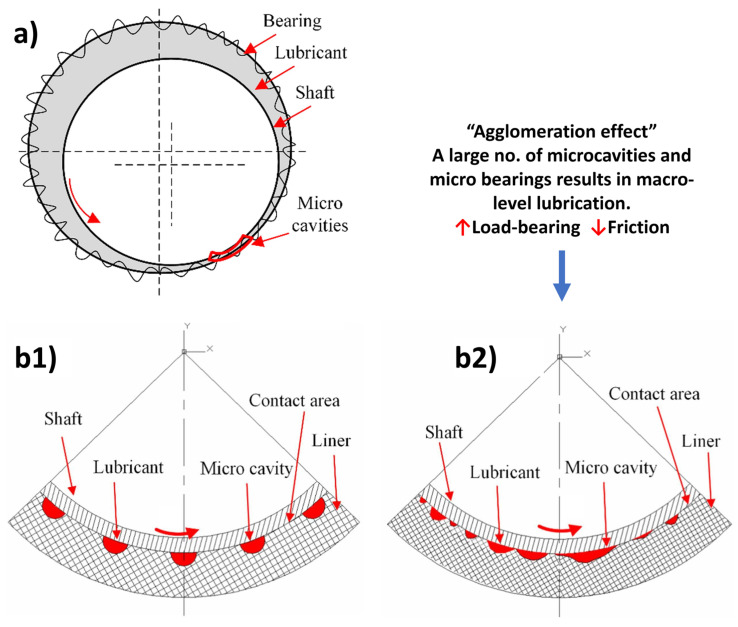
Schematic diagram of (**a**) bearing with rough surface; (**b1**) lubrication regime with fewer microcavities; and (**b2**) lubrication regime with a greater number of microcavities. This results in agglomeration effect and improved bearing life and tribology. Reproduced with permission from Ref. [356].

Bioinspired textures offer significant advantages in the bearing and sealing industry (Figure 24e), particularly for applications requiring long-term maintenance-free operation, such as wave energy converters or offshore triboelectric generators [350,351]. With the increasing emphasis on green tribology, bearings are designed to function in dry, water-based, or biolubricated conditions, often operating under boundary lubrication [352]. This requires surfaces to withstand heavy loads, vibrations, and start–stop cycles of hydrobearings while effectively entrapping debris or retaining lubricants through grooves or textures [353]. Bioinspired surfaces have demonstrated considerable benefits in this regard. Pattnayak et al. [354] developed fish skin-inspired microtextured bearings, showing improved friction, wear, and static and dynamic performance compared to conventional untextured ones. Ji et al. [355] investigated the effect of different surface textures on wettability and film formation during sliding wear of ceramic–steel pairs under fluid lubrication, proposing a model that suggests a bionic-feature tradeoff between the ball and race could customize the friction and wear properties of a bearing system. It can be understood that, under dry friction conditions, bionic texture pits play a crucial role in capturing abrasive particles and debris during friction, effectively reducing sample wear. In wet wear scenarios, these pits serve as reservoirs for abrasive particles and lubricants. The lubricant is dynamically released while capturing abrasives, aiding in wear reduction under boundary and mixed lubrication conditions. Computational fluid dynamics simulations by Xie et al. [356] revealed that surface textures retain lubricants within microgrooves during shaft rotation, enhancing local dynamic lubrication. Agglomeration of numerous micropits and microbearings improves bearing capacity and lubrication performance, as shown in Figure 25. Additionally, the wedge-shaped structures formed by textures create high-pressure zones during fluid flow, further enhancing bearing capacity and surface separation.

### 8.5. Bioinspired Metamaterials

Metamaterials, denoted by the prefix “meta”, indicating “beyond”, represent a novel class of synthetic materials characterized by intricate repetitive patterns at the micro- or nanoscale [357]. In contrast to conventional materials, their properties do not stem from inherent material characteristics but rather from precisely engineered structures or surfaces. These structures govern unique functionalities, such as the manipulation of mechanical stress or tribological stress waves, and are defined by their specific shape, geometry, size, orientation, and arrangement [358].

Metamaterials exhibit remarkable potential in reducing friction and wear, particularly under extreme tribological conditions involving high loads, temperatures, and stress. Reported tribological phenomena associated with metamaterials include adaptive interaction with non-equilibrium environments, surface reorganization, and efficient energy absorption or redistribution [359,360]. For example, Liefferink et al. [361] demonstrated the dynamic friction tuning capability of a kirigami metamaterial surface, enabling external friction adjustment without altering the sliding component. Similarly, Rafsanjani et al. [362] introduced the concept of “tune on-demand” texturing using a kirigami metamaterial, showcasing its application in bioinspired locomotion. They demonstrated the functionality of a bioinspired snakeskin, which, through the cyclic stretching and releasing of its kirigami skin, forms triangular scales when stretched, enabling forward crawling akin to a real snake [362]. The manipulation of scales through stretching and releasing allows for the external control of frictional energy detention or dissipation [362,363,364]. This “on-demand texturing” capability, based on kirigami scales, offers the ability to adjust friction by externally controlling surface roughness [362].

Moreover, friction serves as a crucial energy-absorbing mechanism in various engineering applications, such as automotive brakes, earthquake and bridge vibration absorbers, and prosthetics. Garland et al. [358] devised a honeycomb metamaterial unit cell with internal features capable of sliding past each other during loading and unloading cycles, leading to consistent Coulombic frictional energy dissipation during sliding contact. This technique of frictional energy dissipation serves as a key inspiration for the development of a novel metamaterial, aiming to achieve similar capabilities in dissipating repeated mechanical energy inputs through the movement of internal features.

**Figure 26:**
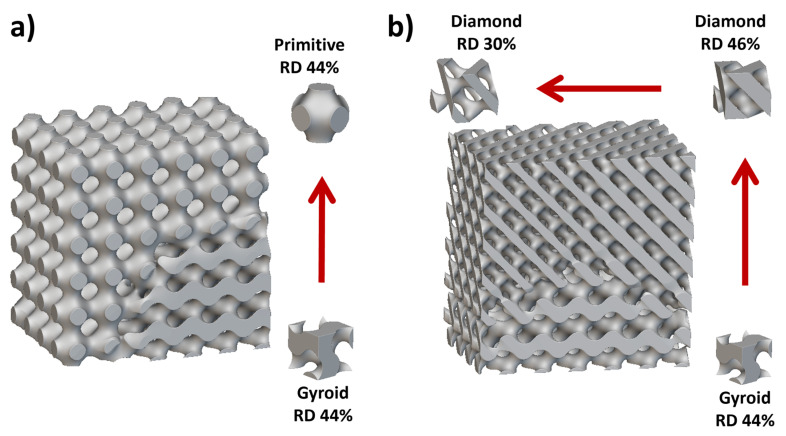
An illustration depicting a multi-morphology metamaterial architecture of triply periodic minimal surfaces (TPMSs), incorporating (**a**) gyroid and primitive unit cells with unit cell grading (printing direction); and (**b**) gyroid and diamond unit cells with unit cell (vertical direction and printing direction) and relative density (transverse direction) grading. The red arrow indicates the grading direction. The cell size for each model is 5 × 5 × 5 mm^3^. The relative density (RD) value is labeled. The model designs are inspired by trabecular bone networks. The 3D models were designed using nTopology software version 4.13.3.

In terms of energy absorption, lattice struts exhibit notable capabilities during impacts due to their ability to undergo plastic deformation or fracture, thus dissipating input energy [365]. Strategically placing features within a metamaterial design can enhance toughness by guiding cracks along predetermined paths. Additionally, grading the lattice structure can enhance energy absorption by ensuring sequential collapse of each layer rather than a single shear band failure [366]. The architecture and base material of unit cells play a crucial role in determining the macroscopic responses of the metamaterial, suggesting that modifying unit cell shape or topology could allow for tailored mechanical performance [367]. Another approach involves incorporating multiple morphologies within a metamaterial structure (Figure 26), with each morphology designed to fulfill specific functions, such as permeability, elongated stress range, elastic modulus, compressive strength, and more [368,369]. For instance, Rezapourian et al. [370] reported that triply periodic minimal surfaces (TPMSs) serve as bioinspired metamaterials due to their resemblance to biological structures like trabecular bone networks (Figure 26). It was shown that the highly interconnected porous structures of TPMS-based metamaterials can achieve improved strength-to-weight ratios, impact resistance, and structural integrity [371]. For example, the TPMS gyroid structure, resembling certain butterfly wing scales, contributes to their structural coloration and antibacterial functionality [367]. Similarly, the TPMS diamond structure mirrors the skeleton of the knobby starfish, featuring remarkable structural gradients and damage tolerance [372]. Meanwhile, TPMS primitive structures, found in sea urchin skeletal plates, are typically optimized (regarding design) and employed for their enhanced strength, flexibility, and delayed failure under impact [372].

## 9. Challenges and Future Prospects in Bioinspired Tribology

**Figure 27:**
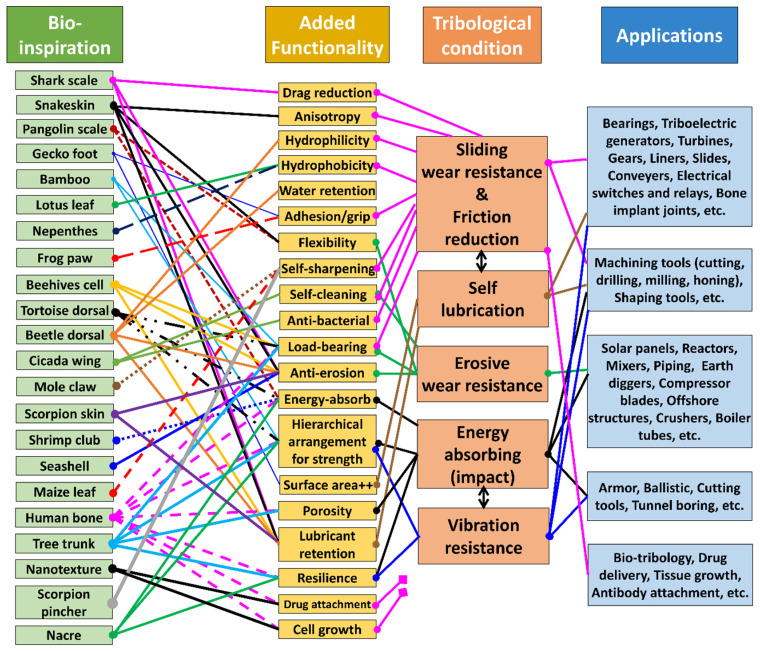
Schematic representation of various bioinspired textures linked to their associated key functionalities, further linked to their possible tribological use conditions and possible application areas. Different colors or styles of interconnected lines are employed to differentiate between bioinspiration types, which are linked to their specific attributes or functionalities, and the tribological conditions that may necessitate corresponding functionalities for specific applications.

The concept of mimicking nature’s design principles to create synthetic materials is gaining traction in tribological research. This approach is expected to remain pivotal in the quest for wear-resistant novel multifunctional materials in the years to come. To enhance comprehension and offer guidance for the initial selection and design of different bioinspired surface textures, Figure 27 was mapped. Figure 27 depicts a general schematic representation of various biologically inspired surfaces/textures along with their associated functionalities, potential tribological conditions of usage, and application areas, as discussed within this review. We think that graphical mapping could be a good tool for engineers and educationists. However, due to the length of information (acquired from published research) and statistical uncertainties regarding some topography, some data may have been mapped as per the authors’ experiences. It is important to note that these biotextures may offer diverse functionalities beyond those mentioned in the schematic and can be customized to suit specific tribological conditions and applied across various domains. Moreover, in several applications, a combination of two or more textures is utilized to enable multifunctionality and superior performance. It is important to acknowledge that nature offers a wealth of features, and there may be additional characteristics that were not included in the graphical representation. The primary objective is to depict the functionalities and desirability of such materials and structures across different tribological scenarios and applications.

Despite promising advancements, biomimicry in tribology remains in its early stages, presenting ample opportunities for further refinement and exploration. For instance, the study of superhydrophobic surfaces, inspired by the lotus leaf effect, has been extensive. Several studies have focused on replicating lotus leaf-like surfaces by combining surface roughness and low surface energy. However, the durability of superhydrophobicity remains a challenge. Recently, drawing inspiration from the Nepenthes pitcher plant, Aizenberg et al. [373] developed slippery liquid-infused porous surfaces (SLIPSs). Unlike conventional lotus leaf-like surfaces where air is trapped by surface roughness, SLIPSs feature pores infused with special liquids to create a solid–water–liquid interface, resulting in excellent repellence to various liquids, along with antifouling, anti-icing, and anti-frost properties, all with high durability, transparency, and even self-healing capabilities. The practical implementation of SLIPSs in tribology holds promise for enhancing the durability of lubricant or chemical-infused surfaces, along with providing additional benefits like antifouling properties. However, their widespread application in tribological systems is still pending realization.

Furthermore, bioinspired materials such as biomaterials must exhibit self-organization under mild conditions of relative motion in the form of debris usage, bone self-structuring, etc. While their sophisticated hierarchical structures (cellular) have never been perfectly replicated using modern technologies, precisely studying self-healing or self-organization behavior, especially at small scales, remains a significant challenge. In contrast, these cellular materials typically lose their function once their structure is destroyed. Therefore, understanding and replicating the dynamic and nanoscale molecular mechanisms of these systems, as well as emulating the adaptation and self-healing abilities of biomaterials under sliding tribology, are crucial.

Generally, biomimetics follows a top-down approach [374], where natural similarities are sought to address a challenge in certain tribological conditions, like the development of an anisotropic low-frictional surface inspired by snakeskin. Conversely, an advanced bottom-up process [375] entails analyzing natural systems to uncover potential tribological and mechanical applications, a field that is underexplored due to limitations in nanomechanical characterization, structural investigation, and manufacturing techniques, resulting in unelicited laboratory studies.

Another topic that requires focus is investigation regarding synergistic effects of multiple bioinspiration features [104]. By examining how various features within biological systems interact and complement each other to achieve specific tribological functions, researchers can gain deeper insights into designing more efficient and effective biomimetic materials and surfaces. For example, understanding how the combination of surface roughness, hierarchical structures, and material/lubricant composition in natural systems contributes to enhanced friction reduction or wear resistance can inspire the development of novel tribological solutions.

Large-scale production of bioinspired materials, especially in achieving simplicity and ambient processing conditions, has been a serious challenge [376]. Attempts to fabricate biomimetic materials often fall short of natural performance as biological materials exhibit multifunctionality. Replicating such multifunctionality remains a challenge. For instance, designing cellular materials inspired by nacre or bone or tree trunk, etc., involves incorporating microcapsules or vascular networks with transport/healing agents to enable spontaneous recovery from damage (self-healing).

Improvements in biomimetic designs through utilization of modeling hold imminent importance as they enable exploration of a wide range of design parameters, such as surface topography, material properties, and environmental factors, to optimize the performance of bioinspired tribological systems [198,377,378]. Furthermore, modeling enables us to gain insights into the underlying mechanisms governing tribological behavior in systems, leading to the development of more realistic and effective biomimetic designs. Although biological systems advocate the use of hierarchical multiscale surface textures, most of the published experimental and numerical works have mainly addressed effects induced by single-scale surface textures [379,380]. Multiscale surfaces offer advantages in high-load, low-speed conditions by reducing real contact area, supplying additional lubricant, and trapping wear particles. They also increase hydrodynamic pressure and provide a greater reservoir volume for lubricant and wear debris in mixed lubrication. Moreover, smaller densely distributed textures improve wetting behavior and lubricant distribution, reducing cavitation and flow circulation to enhance load carrying capacity. However, further fundamental studies are required to evaluate essential parameters in multiscale textures, including aspect ratio, area density, and their relationship to texture size, to optimize their tribological behavior. Additionally, machine learning algorithms have been increasingly utilized to analyze large datasets and identify optimal texture designs for improved tribological performance [381,382].

**Figure 28:**
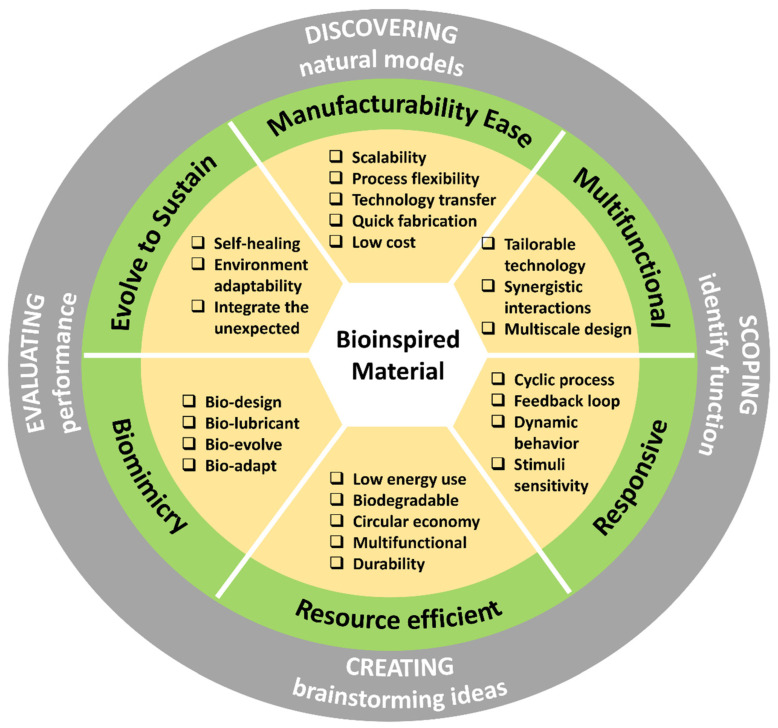
Circle of a bioinspired tribological material. Six key necessities (green) of a bioinspired tribological material are shown, i.e., biomimicry, evolve to sustain, manufacturability ease, multifunctionality, responsive, and resource-efficient. Under these necessities are their key drivers (yellowish). The outer periphery (gray) marks the guiding principle behind a typical biomimetic approach, i.e., discover natural models, identify function within material, create materialistic approach, and evaluate material’s performance.

Another essential step in futuristic growth in bioinspired tribology is the utilization of materials-property databases in terms of comprehensive repositories of material properties, including tribological features [383,384]. Through data-driven approaches and computational modeling, materials-property databases enable the prediction and exploration of material behaviors under different conditions, facilitating the design of bioinspired surfaces with tailored tribological characteristics. Moreover, these databases facilitate knowledge sharing and collaboration within the scientific community, accelerating the pace of innovation in bioinspired tribology. Figure 28 shows a general circle of a bioinspired tribological material.

**Figure 29:**
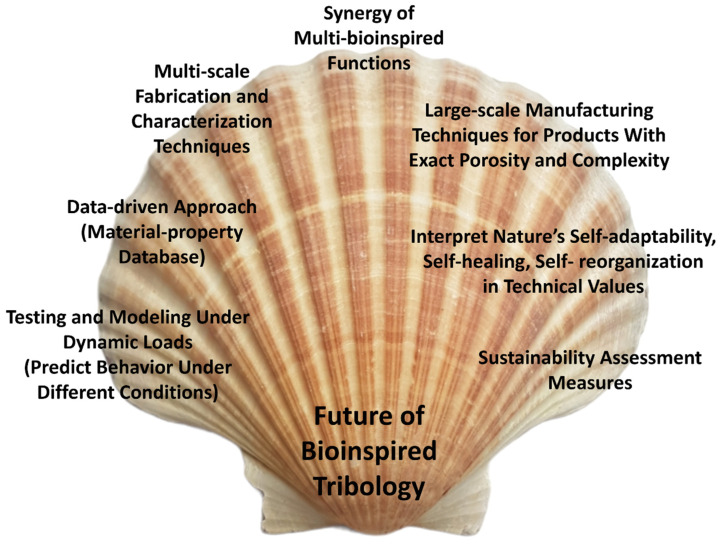
The future of bioinspired tribology advancement demands integrating key aspects of modeling and testing, data-driven approaches, novel multiscale fabrication and characterization techniques, synergy of multi-bioinspired functionalities, new manufacturing methods to fabricate intricate geometries of bioinspired materials, approaches towards interpreting nature’s science, and measures towards sustainability assessment.

In general, the advancement of bioinspired wear-resistant materials depends on comprehending structure–composition–property relationships, building a comprehensive database for customizable design and optimization, as well as employing advanced manufacturing techniques to meet complex design requirements. Advancements in additive manufacturing enable the printing of a wider range of materials and the creation of hierarchical and composite structures [385]. Additionally, 4D printing can lead to integration of biological materials into synthetic components towards the development of novel bioinspired materials with superior properties [386,387]. Furthermore, establishing relationships between quasistatic and dynamic testing, as well as advancements in testing and modeling of bioinspired materials under dynamic loads, are crucial for understanding material responses and optimizing wear resistance [13,251]. Investigating the effects of multiple structural elements (multi-bioinspired) acting in synergy and understanding the role of self-healing and structural hierarchy on wear resistance are also essential for future progress. Finally, optimal utilization of materials, porosity, and fluids for superior lubrication, heat transfer, etc., is of interest in numerous applications [53,388,389]. Apart from this, the creation of efficient databases and computational models supported by a machine learning approach for ease in technology transfer will aid in broadening the range of knowledge and collaboration [382,384]. By addressing these key areas, bioinspired tribology can advance significantly, leading to the development of durable, efficient, and sustainable materials and structures with enhanced tribological properties. Figure 29 shows the aspects to be integrated for advancements in bioinspired tribology.

## 10. Summary

Drawing from the virtually limitless wellspring of bioinspiration, natural materials exhibit remarkable mechanical efficiency, offering a wealth of features, such as self-optimized microstructures, exceptional load-bearing ability, hierarchical arrangements, and environmental adaptability, etc. These attributes, combined with wear resistance and the ability to modulate stiffness and friction in response to external stimuli, provide a rich source of inspiration for developing advanced sustainable tribological materials. As bioinspired tribology evolves, a multidimensional approach encompassing design, manufacturing processes, and performance evaluation becomes imperative. By examining successful examples from nature, distinct patterns in microstructural organization become evident, leading to the classification of design elements crucial for wear resistance in biological materials under various conditions, such as sliding, erosion, impact, or machining. Moreover, the current study objectifies and delves into the common traits of biological materials essential for specific tribological applications, considering factors like self-organization of microstructures, self-cleaning, flexibility, hydrophobicity, and antibacterial properties, etc. Looking ahead, advancement regarding bioinspired wear-resistant materials hinges on understanding structure–composition–property relationships, establishing comprehensive databases for customizable design and optimization, and employing advanced manufacturing techniques to meet intricate design needs. Innovations in additive manufacturing, including 4D printing, offer opportunities for integrating biological materials into synthetic components, paving the way for novel bioinspired metamaterials with superior mechanical and tribological properties. Additionally, progress in testing and modeling of bioinspired materials under dynamic loads, as well as investigating the synergistic effects of multiple structural elements and understanding the role of self-healing and structural hierarchy, are crucial for future advancements. Optimal utilization of materials, porosity, and fluids for superior lubrication and heat transfer, alongside the creation of efficient databases and computational models, will facilitate technology transfer and collaboration, expanding the scope of knowledge in this field. By addressing these key areas, bioinspired tribology can significantly progress, leading to the development of durable, efficient, and sustainable materials and structures with enhanced tribological properties.

## Figures and Tables

**Table 1:** Multifunctional features required in different tribological materials/situations [13,24,35,36,37,38,39,40,41,42,43,44,45,46,47,48,49,50,51,52,53,54,55,56,57,58,59,60,61].

Applications	Key Functionality Required	Key Tribological Modes
Wind turbines and propellers	Anti-icing, Antifouling, Self-cleaning, Erosive resistance, Lightweight, Drag-resistance, Hydrophobicity	Sliding and Leading-edge erosion
Bearings, seals, actuators, gears, and shafts	Flexibility, Compactness, Self-lubrication, Adaptive response, Load carrying capability, Environmental adaptation, Vibration damping, Noise reduction, Friction reduction, Hydrophobicity	Sliding
Rail tracks, wheels, and braking systems	Self-healing smart materials, Self-adaptive wheels, and brakes (friction) to changing loads, speed, etc., Energy harvesting from vibration damping, Self-cleaning, Wear resistance	Sliding, Rolling
Piston rings, cylinder liners, camshafts	Self-lubrication, Adaptive response, Wear resistance, Heat dissipation, Friction reduction, Self-cleaning, Vibration damping, Noise reduction	Sliding
Cutting tools in machining, drill bits	Self-sharpening, Wear resistance, Heat dissipation, Anti-adhesion chip control, Lubrication enhancement, Adaptive control, Hydrophobicity	Sliding, Impact, Abrasion, Erosion
Prosthetic joints, dental implants, and surgical instruments	Biocompatibility, Antibacterial, Osseointegration, Flexibility and Range of Motion, Self-lubrication, Load carrying capability, Surgical precision, Responsive material to adapt tissue changes	Sliding, Impact, Tribocorrosion, Quasistatic compression
Ship hulls, hydrofoils, and swimsuits	Drag resistance, Antifouling, Hydrophobicity, Flexibility and maneuverability, Hydrophobicity, Air-filled cavities, or flexible fins for buoyancy	Hydro-drag, Erosion, Corrosion
Spatial satellites	Erosion and impact resistance from space debris, Adaptive or morphing structures, Energy harvesting solar panels, Radiation resistance, Bioinspired locomotion	Erosion, Impact
Material handling equipment (crushers, screens, conveyor systems, hoppers, chutes, etc.)	Erosion and impact resistance, Efficient material flow patterns, Abrasion resistance, Self-cleaning of debris or material build-up, Flexible components for easy throughput of feedstocks	Abrasion, Erosion, Impact
Pumps (impellers, casing, suction/discharge nozzles, etc.) and pipelines	Drag reduction, Self-cleaning, Improved fluid handling, Antifouling, Anti-corrosion	Abrasion, Erosion, Corrosion, Cavitation
Hydro turbines and boilers	Adaptive blade design, Efficient heat transfer, Drag reduction, Cavitation resistance	Slurry erosion
Plows, tillage tools	Self-sharpening, Self-cleaning, Hydrophobicity, Efficient soil penetration, and tillage	Erosion, Abrasion, Impact
Ski edges, snowboards, and bicycle chains	Hydrophobicity, Anti-icing, Reduced friction, Enhanced grip adhesion, Resilience, Shock absorption, Lightweight	Abrasion, Sliding
Solar panels	Self-cleaning, Erosion resistance, Flexibility, Enhanced light harvesting surface	Solid particle erosion
Airframe structures	Lightweight, High strength, Flexibility, Natural sound-damping (owl wings-inspired), Vibration stability and maneuverability, Drag reduction	Air drag/friction, Trailing edge erosion
Cricket bats, golf clubs, and hockey sticks	Lightweight, Shock absorption, High strength, Resilience, Drag reduction	Impact, Air drag
Automotive body, crash barriers on highways, and military armor	Energy absorption, Impact resistance, Flexibility for movement, Adaptive camouflage	Impact
Bulldozers, hydraulic hammers, and excavator teeth	Impact resistance, Self-sharpening edges, Material anti-adhesion, Erosion resistance, Abrasion-resistant, Hydrophobicity	Impact, Abrasion, Erosion
Bone implants for hip, knee, skull, etc.	Energy absorption, Lightweight, Porous, High strength, Adaptive lubrication, Antibacterial, Cell attachment	Sliding, Impact, Quasistatic, and dynamic compression
Robotic grippers, Microbots, bio-MEMS/NEMS	Adaptive adhesion, Variable stiffness, Biocompatibility, Friction anisotropy, Antibacterial	Sliding, Frictional grip

**Table 2:** Results of different bioinspired structures under sliding wear conditions as reported in the literature. It is important to highlight that the absence of key tribotechnical parameters in many published findings prevents calculation of resulting tribological stress conditions. However, instances of tribocontact stress calculations have been observed assuming Hertzian contact and no wear to the counterbody in a few studies.

Bioinspired Texture and Material	Sliding Test Parameters	Friction Coefficient (CoF)	Conclusions/Texture Effect
Snake/Lizard scales-inspired (100Cr6) by laser texturing [124,126]	Velocity: 0.02–0.17 m/s Load: 2 N Pin-on-disc Counterbody: 100Cr6	~0.3 dry ~0.01 lubricated	50% low CoF in dry condition. 80% low CoF in lubricated condition for faster sliding speeds, i.e., >70 mm/s.
	Velocity: 0.02–0.17 m/s Load: 2 N Pin-on-disc Counterbody: PEEK	~0.12–0.18 dry ~0.1 lubricated	30–50% low CoF in dry condition. 40% low CoF in lubricated condition.
	Velocity: 0.02–0.17 m/s Load: 2 N Pin-on-disc Counterbody: Alumina	~0.6–0.9 dry ~0.05 lubricated	Reduction in friction strongly depends on the sliding speed. Low CoF (20%) at slow speed in dry condition. Low CoF (70%) at faster speed in lubricated condition.
Dung beetle-inspired multi-hierarchical micro/nanotextures (AISI 440C steel) by Laser ablation and coating technology [143]	Test: 15 min Load: 0.5, 1, 2, 4 N Hertzian contact pressure: 378–756 MPa Frequency: 3, 6, 9, 12 Hz Stroke: 0.004 m Ball-on-disc Counterbody: Steel ball Lubricant: Water	Decrease in CoF (~0.25) with increase in load or frequency for both single- and multi-bioinspired textures due to generation of high hydrodynamic pressure.	Possibility of developing superhydrophobic micro/nanostructures. Role of superhydrophobic in enhancing wear resistance. More than 50% wear rate reduction using multi-bioinspired textures than single-bioinspired.
Snake scales-inspired (AISI 52100) by Photochemical machining process [110,125]	Velocity: 1 m/s Load: 10 N Pin-on-disc Counterbody: AISI H13 tool steel pin	CoF Reduction of 37.5% was obtained for sliding parallel to lengthwise scales direction.	Anisotropic friction behavior observed as a function of the sliding direction. Friction depends on aspect ratio (scales) and percentage of texturized area on the surface.
Snake scales-inspired (Silicon/Epoxy) by Direct moulding [136]	Velocity: 0.002 m/s Load: 0.5–3.92 N Disc-on-disc Counterbody: Same as samples	~0.1–0.3 dry	Complex friction behavior may lead to the possibility of inversion of friction anisotropy depending on the materials’ stiffnesses, the aspect ratios of the structural features, and substrate roughness.
Fish scale-inspired(MXene Ti_3_C_2_T_x_ based fabric composites) by Impregnation and drying [146]	Velocity: 0.7–1 m/s Load: 25–40 MPa Pin-on-disc Counterbody: AISI-1045 steel pin	~0.04 dry	77% lower wear rate compared to pure fabric composite. 38% lower CoF compared to pure fabric composite. CoF and Wear rate increase with increasing sliding speed. Fish scale-like structure strengthens the mechanical interlocking and chemical bonding between matrix and MXenes.
Fish scale-inspired (Mold steel) by 3D Printing [147]	Velocity: 0.084–0.134 m/s Load: 5–25 N Pin-on-disc Counterbody: Bronze pin (HRC 45) Lubrication: Pharmaceutical white oil	~0.10–0.18 lubricated, tilt 45°	Tilt 45° resulted in the lowest CoF value. Squeezing out of lubricant observed. CoF decreases with the increase in sliding velocity. CoF decreases with the increase in textures number. CoF increases with the increase in sliding load.
Ocellated Skink Scale-inspired (polydimethylsiloxane) by 3D Printing [148]	Velocity: 0.002 m/s Load: 0.02, 0.05, 1 N Stroke: 0.004 m Ball-on-disc Counterbody: Chrome steel ball	~1.04 for 0.2 N, dry ~0.93 for 0.2 N, dry ~1.04 for 1 N, dry	For the sliding loads 2 and 0.5 N tests, the CoF reduced by 30% and 26%, respectively. Average wear tracks widths were reduced with 61%, 48%, and 44% reduction under 0.2 N, 0.5 N, and 1 N loading conditions, respectively.
Pangolin scale-inspired (obtained from real animal) mounted on steel block [149]	Velocity: 0.42 and 0.84 m/s Load: 30–90 N Distance: 252 m Block-on-ring Counterbody: 0.04 m gray iron ring	~0.22–0.42 for 0.84 m/s dry ~0.28–0.49 for 0.42 m/s dry	The wear rate at higher velocity was larger than that at the lowest velocity. Transfer film at 0.84 m·s^−1^ velocity was enlarged a little as compared with that at 0.42 m·s^−1^ velocity. High loads led to lower CoF.
Lotus leaf-inspired nano pillars coated with PMMA (silicon wafers/PMMA) by Soft lithography [101]	Velocity: 2 μm/s, 0.001 m/s Load: 0–80 nN, Time: 15 min Ball-on-flat Counterbody: 1.25 μm Borosilicate ball	~0.025 untextured, dry ~0.01 textured, dry	Patterns show superior adhesion and friction behavior due to their hydrophobic nature and reduced contact area. Patterns show lower CoF than PMMA thin film owing to reduced contact area.
Lotus leaf-inspired micropillars coated with PMMA (silicon wafers/PMMA) by Soft lithography [101]	Velocity: 0.001 m/s Load: 0.003 N, Time: 15 min Ball-on-flat Counterbody: 1.25 μm Borosilicate ball	~0.65 untextured, dry ~0.2 textured, dry	Patterns show lower CoF than PMMA thin film owing to reduced contact area.
Lotus leaf-inspired circular and bi-triangular microdimples by 7 and 20% density (100Cr6) by Lasers [128]	Velocity: 0.2, 0.6, 1 m/s Load: 15 N Scar dia: 0.008 m Pin-on-disc Counterbody: 100Cr6	~0.56 for 7% and 20% dense dimples, 1 m/s, bi-triangular textured dimples ~0.55 for 20% dense dimples, 1 m/s, circular textured dimples	Dimples with higher density (20%) showed less wear and lower friction compared to untextured disks or disks with 7% dense dimples, especially at higher speeds. CoF in sliding pair with a textured sample is less than untextured one. Higher dimples increase the entrapment of a larger number of wear particles
Maize leaf-, Shark-, Snake-, Pitcher-, and Lizard skin-inspired surfaces (PLA) by FDM 3D printing [134]	Velocity: 0.01 m/s Distance: 200 m Load: 27, 59 MPa Time: 15 mins Stroke: 0.01 m Ball-on-plate Counterbody: 0.01 m AISI 52,100 balls Sliding directions: high friction direction (HFD) and the low friction direction (LFD)	At 27 MPa four times rise in the wear track width was reported when sliding against the anisotropic patterns. Lowest and highest values of wear track width (anisotropic) were found for pitcher and snake patterns, respectively. Lowest and highest values of wear track width (isotropic) were found for flat and shark patterns, respectively. Larger wear track width when increasing the contact pressure to 59 MPa. Both the bioinspired pattern and the sliding direction could be advised as the optimum test parameters for reaching the target frictional behavior.	The surface texture exhibited a considerable effect on friction anisotropy under a contact pressure of 27 MPa, specifically for the lizard, the snake, and the pitcher plant. Anisotropic pattern and sliding direction (LFD) could be advised as the optimum test parameters for reaching the best frictional behavior. The CoF decreases outstandingly when the contact pressure increases up to 59 MPa irrespective of the used patterns and particularly when sliding in the HFD due to the increased contact pressure; the morphology of the 3D-printed surface is damaged and compressed against the worn surface, causing the rise in surface temperature.
Tree frogs’ toe pad-inspired elastic micropatterned dimple/pillar arrayed structures for wet sliding contacts (Polydimethylsiloxane elastomers) by FDM 3D printing [132]	Velocity: 0.003–0.2 m/s Load: 0.95 N Time: 15 mins Lubrication: Deionized water Pin-on-disc Counterbody: Spherical pins and disks made from Polydimethylsiloxane elastomers	High densities of microstructures are conducive to achieving stable friction from low to high sliding speed. Flat disk displayed higher friction than the microstructured disks at low speeds (v < 0.04 m/s). In the high-speed region (v > 0.04 m/s), all the friction forces were well above those of the flat disks.	Three types of patterns: round dimple, round pillar, and hexagonal pillar. Surface with microdimples displayed a reduction in friction with an increase in the pattern area density. The area density of pillar patterns had no significant effect on the friction property at low sliding speeds, whereas it became a dominating factor with the increase in sliding speed.
Lotus leaf-inspired micropillars/channels coated with DLC or Z-DOL (silicon wafers/DLC/Z-DOL) by Photolithography and Deep Reactive Ion Etching [150]	Velocity: 0.001 m/s Load: 0.003 N Time: 15 mins Ball-on-flat Counterbody: 0.001 m soda lime balls	~0.5 for bare, untextured Si wafers ~0.15 for bare Si wafers, pillar-textured ~0.2 for DLC coated, untextured ~0.11 for DLC coated, pillar-textured ~0.15 for Z-DOL coated, untextured ~0.09 for Z-DOL coated, pillar-textured	Chemical modification by coating DLC/Z-DOL and topographical modification by micropatterns on Si surfaces lowers friction and wear. Topo-modification of Si surfaces renders a reduction in CoF due to the physical reduction in the contact area and debris removal. Micropillars show lower friction than microchannels due to lower contact area. A dual/combined surface modification significantly lowers friction and shows no observable wear of either material.
Lotus leaf-inspired circular microdimples (Ti-alloy coated with CrN-MoS_2_-Ag) by Lasers and Sputtering [151]	Frequency: 20 Hz Load: 20, 30 N Stroke length: 0.001 m Ball-on-disc Counterbody: 10 mm SiC ball	~0.2, untextured and textured coated surface More than 50% reduced wear for textured coated surface in comparison to untextured coated surface.	Significant 74.4% and 60.4% improvement in wear resistance compared to substrate surface under sliding loads of 20 and 30 N. Dimples resulted in secondary lubrication and capture of debris.
Circular microdimples on bronze surface (CuSn6) by Macro indenter [133]	Velocity: 200 r/min Load: 5, 15, 25 N Scar dia: 0.008 m Ball-on-disc Counterbody: 0.004 m Graphite balls	~0.26, 0.22, 0.20 at 5, 15, 25 N for textured. ~0.35, 0.26, 0.24 at 5, 15, 25 N for untextured.	15%, 21%, 12% decrease in wear rate for textured samples after 5 N, 15 N, 25 N sliding.
Seashell-inspired composite surface pattern (Ni3Al matrix with Sn and Ag) by LMD and wire cutting [142]	Load: 25 N Amplitude: 5 mm Frequency: 1 Hz Sliding time: 1800 s Ball-on-disc Counterbody: 0.006 m high-carbon steel balls	Increasing texture density results in CoF and wear rate decrease first (until 20%) and later leads to increase in their values for textures without SL. For textures with SL, the decrease in their values was noted all throughout from 0–50% density of textures.	Textured surfaces with solid lubricants were always better than patterned without solid lubricant due to the wear debris entrapment of solid lubricants and could improve the anti-friction performance.
Mussel-inspired copolymer (MPC with surface induced Nanodiamonds by Copolymerization and stirring [152]	Load: 25 N Amplitude: 0.005 m Frequency: 1 Hz Sliding time: 1800 s Ball-on-disc Counterbody: 0.006 m high-carbon steel balls	~0.028 for non-lubricated textured surface. ~0.017 for ND-lubricated textured surface.	Significantly reduced the wear on the tribopairs but also further decreased the COF by approximately 40%. ND could be attributed to the rolling effect of the nanoparticles.
Honeycomb-inspired self/lubricating steel composite (AISI 4140 steel/SnAgCu–TiC) by Laser cladding [137]	Velocity: 200 r/min Load: 20 N Block-on-ring Counterbody: AISI 4140 steel	Compared with AISI 4140 steel, the average COF of self-lubricating textured composites was decreased by 67%, and the wear depth was decreased by 42%.	Self-repairing behavior of bionic textured AISI 4140 steel filled with multi-solid lubricants. Texturing enables wear debris trapping. Wear debris consists of solid lubricants.
Frog paw-inspired self/lubricating steel composite (AISI 4140 steel/SnAgCu) by Additive manufacturing [138]	Velocity: 0.016 m/s Load: 30 N Block-on-ring Counterbody: 0.063 m Si_3_N_4_ ceramic ball	Compared to untextured AISI 4140 Steel, AISI 4140-SnAgCu with optimized bionic texture reduced the average friction coefficient by 20%, fluctuation degree by 54%, and wear track depth by 65%.	Optimized parameters of bionic hexagonal type microtexture were reported. Optimized surface texturing resulted in uniformly dispersed lubricants on the worn surface. Superior friction and wear reduction after optimization.
Biomimetic coating (Calcium phosphate on Ti) by Biomimetic mineralization [153]	Velocity: 0.02–0.17 m/s Load: 2 N Pin-on-disc Counterbody: 100Cr6 pin Under dry and simulated body fluid.	~0.6 dry for pure Ti ~0.3 dry for coating ~0.01 lubricated for coating	50% lower CoF in dry condition in comparison to pure Ti. 80% lower CoF in lubricated condition for faster sliding speeds, i.e., >0.07 m/s.
Cancellous bone-inspired ZrO_2_ coating (ZrO_2_/modified PTFE) by Thermal spraying/Laser texturing [77]	Velocity: 0.01–0.15 m/s Load: 1–30 N (654–2035 MPa) Ball-on-disc Counterbody: ZrO_2_ ball, 0.005 m dia. Dry sliding.	Low CoF (<0.065) at ultrahigh contact pressure (10 N, 1411 MPa) with an extremely long lifetime (>1 × 10^6^ cycles). CoF decreased from 0.070 to 0.048 as the load increased from 1 to 30 N. CoF decreased from 0.089 to 0.048 when the sliding velocity increased from 1 to 0.15 m/s.	Low CoF maintained a large range of sliding velocities and applied loads. Textured composites show zero-wear properties.
Biomimetic metal ceramic composite (Ti/Ti64 alloy and Calcium phosphate) by Laser deposition [154]	Stroke length: 0.01 m Stroke rate: 0.017 m/s Load: 5 N Ball-on-disc Counterbody: 100Cr6 ball Under simulated body fluid	~0.85–0.9 SBF lubricated	CaP addition to the Ti increased the strength, hardness, and wear resistance. Wear rate decreased by 92% when 10% wt CaP was added to CP-Ti. 5% wt CaP to Ti64 decreased the wear rate by 70%.

**Table 3:** Results of various bioinspired topographies on biomaterials for sliding wear conditions, as reported in the literature.

Bioinspired Texture	Biomaterial	Topography/Texture Feature	Functionalities
Lotus leaf-inspired [102,164,172]	Polymethyl methacrylate (PMMA) structures	Hierarchical surface featuring protrusions and valleys ranging from 3–10 µm, with nanometric particles (70–100 nm) of a hydrophobic wax-like material in the protrusions.	Antibacterial, self-cleaning
Sharkskin-inspired [165,166]	Polydimethylsiloxane embedded elastomer	Denticles refer to diamond-shaped scales with a raised ridge and concave groove, exhibiting some nanostructures. The Sharklet model comprises rectangular features.	Decrease in bacterial adhesion, whether independently or in combination with other chemical and photocatalytic substances
Cicada wings-inspired [109,173,174]	Poly (ethylene terephthalate), Titanium, Silicone-based elastomer nanopillars	Highly organized array of nanosized pillars or cones of varying dimensions.	Antibacterial
Rice leaves-inspired [156]	Polypropylene (PP)	Papillae in micron height on the surface.	Antibacterial, 53% reduction in bacterial adhesion.
Sea urchin-inspired [175]	Poly-lactic acid (PLLA)	Spiny finger-like structure.	Antibacterial, selfcleaning
Gecko skin-inspired [161]	Gecko skin	Dome-shaped scales are organized in a hexagonal pattern. These scales range from 100 to 190 µm in diameter and approximately 50 µm in height at the back. Spinules, resembling hairs reach up to 4 µm in length, with sub-micron spacing and a small radius of curvature usually ranging from 10 to 20 nm.	Antibacterial
Tree frog toe-inspired [160,176]	Polystyrene/Polydimethylsiloxane composites, Hydrogel	Hexagonal cells, separated by channels.	Improved adhesion
Butterfly wing-inspired [165]	Polydimethylsiloxane embedded elastomer	The wing surface features microscales, parallel ridges, and tile-like microstructures, along with nanoscale ribs and lamella-stacking nanostripe structures.	Self-cleaning coatings,
Nanoholes with atomic vacancies [177]	MoS_2_	Nanohole-enabled nanomaterials	Anti-infection, enhanced biofilm formation.
Nanospikes [178]	Polypeptide-functionalized titania nanospikes	Titania (TiO_2_) nanospike coating on the surface of a Ti substrate.	Antibacterial
Rose petals-inspired [100,179]	Polyethylene terephthalate glycol modified substrates	Hierarchical structures feature micropapillae.	Antibacterial and cell attachment.

**Table 4:** Results of different bioinspired structures under erosive wear conditions, as reported in the literature.

Bioinspired Texture and Material	Erosion Parameters	Erosive Wear Rate	Conclusions/Texture Effect
Desert scorpion skin-inspired V-groove surface (AA6061 alloy) by Wire cut EDM [189]	Impact velocity: 30 m/s Erodent: SiC (irregular) Particle diameter: 100–150 μm Mass flow rate: 0.025 g/s Impact angle: 30° Nozzle diameter: 8 mm Impact distance: 200 mm Erosion time: 600 s	Stabilized erosion rates (200–600 s) for Textured: 4 × 10^−8^ g/mm^2^·s Untextured: 9 × 10^−8^ g/mm^2^·s	Anti-erosion property of the textured specimens increased by approximately 57.4%. The surface area due to V-shaped texture was increased by 67% compared to that of the smooth specimen. V-shaped textures changed the angle at which the erodent was incident to 90°, leading to reverse air flow and reduced erodent kinetic energy and erosion rate.
Desert scorpion skin-inspired V-groove surface (ABS polymer) by Simulation study [212]	Erodent: SiC (irregular) Solid particles density: 3.2 g/cm^3^ Particle diameter: 105–830 μm Impact angle: 30°, 60°, 90° Erosion time: 10 s	Simulation by finite element software was applied to predict (regression analysis) the erosive wear behavior.	Bionic surface morphology can change the impact angle between particle and target and reduce the probability of impact angle of 20–0 to some extent and hence reduce erosion rate. Improved erosion resistance by 25–28% for textured samples for best conditions.
Desert scorpion skin-inspired Square, V, U grooved fan blades (Q235 steel) by Wire cut EDM [190]	Impact velocity: 30 m/s Erodent: SiO_2_ (irregular) Particle diameter: 150 μm Mass flow rate: 0.025 kg/s Impact angle: 30° Nozzle diameter: 150 mm Nozzle length: 1000 mm Impact distance: 60 mm Erosion time: 450 s	Stabilized erosion rates (230–450 s) for Square-groove: 18 × 10^−8^ g/mm^2^·s Square-groove: 15 × 10^−8^ g/mm^2^·s U-groove: 12 × 10^−8^ g/mm^2^·s V-groove: 12 × 10^−8^ g/mm^2^·s	Blades with optimum parameters could effectively improve anti-erosion property by 29%. The formation of “air cushion” equips the V-type groove bionic surface with the best erosion resistance compared with other samples. V-groove surface, feature size of 4 mm, and distance of 2 mm
Androctonus australis skin-inspired V-groove, convex hull, and hexagonal pit grooved surface (Stainless steel) by 3D printing [186]	Impact velocity: 25 m/s Erodent: SiO_2_ (irregular) Particle diameter: 260–941 μm (varied) Mass flow rate: 0.025 kg/s Impact angle: 30° Impact distance: 21 cm Erosion time: 350 s	Stabilized erosion rates (90–350 s) for Untextured: 10 × 10^−7^ g/mm^2^·s V-groove: 9 × 10^−7^ g/mm^2^·s V-groove + convex hull: 8 × 10^−7^ g/mm^2^·s V-groove + hexagonal pit: 8 × 10^−7^ g/mm^2^·s V-groove + convex hull + hexagonal pit: 7 × 10^−7^ g/mm^2^·s	Hexagonal pit structure had best anti-erosion effect. The reason is that hexagonal pit slot can form the rotational flow, which can affect the motion direction of the particles and reduce impact velocity of the particles. A combination of groove + convex hull + large depth of hexagonal pit led to lowering the erosion rate.
Fish- and Snakeskin-inspired flexible structures (Ti6Al4V + rubber) by 3D printing [24]	Impact velocity: 30, 50, 80 m/s Erodent: SiO_2_ Particle diameter: 0.1–0.6 mm Impact angle: 30°	10, 22, 96 mm^3^/kg at 30 m/s, 50 m/s, 80 m/s.	Fish- and snake scale-inspired structures showed improved wear at 50 m/s and a slightly higher wear rate at 30 and 80 m/s compared to rubber.
Mole pelt-inspired flexible structures (AISI316L + Diamond + rubber) by 3D printing [24]	Impact velocity: 30, 50, 80 m/s Erodent: SiO_2_ Particle diameter: 0.1–0.6 mm Impact angle: 30°	2, 16, 80 mm^3^/kg at 30 m/s, 50 m/s, 80 m/s.	Mole pelt-inspired flexible structures exhibited around two times better erosion resistance during 30 m/s impact velocity compared with Hardox 400 and polymer while being compressed by 50% of its initial thickness without scarifying its wear resistance.
Dung beetle-inspired micro/macro surface (Z-ABS) by 3D printing [211]	Impact velocity: 56 m/s Erodent: Al_2_O_2_ Particle diameter: 212–300 μm Mass flow rate: 5.7 g/s Impact angle: 0–90° Impact distance: 20 mm Erosion time: 10 s	Erosion rate (g/g): 2.1 × 10^−4^ at the impact angle of 15°, 3.6 × 10^−4^ at impact angle of 30°, and 1.2 × 10^−4^ at impact angle of 90°. The erosion rate is 3.5 × 10^−4^ at impact angle of 45°.	Erosion rate of macrotextured surface in parallel (0°) direction was approximately 28% higher than microtextured surface. Erosion rate of macrotextured surface in perpendicular (90°) direction was approximately 71% lower than microtextured. Air cushion effect could be another mechanism of anti-erosive activity of the textured surface. Rebounded particles in perpendicular (90°) led to decreased incoming impact and final wear rate.
Tamarisk-inspired micro-V-groove surface (Original tamarisk trunk) [55]	Impact velocity: 30 m/s Erodent: SiO_2_ Particle diameter: 40–70 mesh Mass flow rate: 25 g/s Impact angle: 90° Impact distance: 20 mm Erosion time: 10 s	Erosion weight loss (mg): Square groove: 93 mg U-type groove: 91 mg V-type groove: 75 mg	V-groove surface on centrifugal fan blades with optimum parameters can effectively improve anti-erosion property by 28.97% in comparison to other, i.e., with square groove surface, U-type groove surface, and convex dome surface, and the sample with smooth surface.
Tamarisk-inspired micro-V-groove surface (acrylic plate) [194]	Impact velocity: 30 m/s Erodent: SiO_2_ Particle diameter: 230 µm Mass flow rate: 2.83 g/s Impact angle: 10°, 90° Impact distance: 20 mm Erosion time: 1 h	Erosion weight loss depends on global and local impingement angle.	Cylindrical surfaces with grooves are shown to be more resistant to erosion when compared to smooth surfaces, regardless of the wind direction. Erosive wear rate range of global impingement angles of a grooved surface changes according to the ductility of the target surface. Factors such as multiple impacts of particles and the air swirls within grooves were shown to exert negligible effects on erosion.
Scorpion skin-inspired bumped, grooved, and curvature structures (Stainless steel) by 3D printing [188]	Impact velocity: 30 m/s Erodent: SiO_2_ Particle diameter: 230 µm Mass flow rate: 2.83 g/s Impact angle: 30° Erosion time: 180 s	Curved surfaces with bumps and grooves show a higher erosion rate than just curved surface. Smooth surfaces with bumps and grooves show lower erosion rate than just smooth surface.	Anti-erosion property due to bumps can be enhanced by 25%. Bumps have the better anti-erosion performance due to reduced area of impact and enlarge relative impact angle in a certain area, and, the smaller the relative impact angle, the lower the erosion rate.

**Table 5:** Results of different bioinspired structures under impact-absorbing (wear) conditions, as reported in the literature.

Bioinspired Structure	Impact/Mechanical Tests	Conclusions/Texture Effect
Pangolin scale (obtained from real animal) [135]	Microindentation: Load of 1 N holding for 15 s. Tensile testing: strain rates from 10^−5^/s to 10^−1^/s Compression testing: 10^−3^/s	Microhardness around 220 MPa. Similar tensile behavior when stretched longitudinally and transversely, Young’s modulus around 1.5 GPa, and tensile strength about 108 MPa. Able to absorb large energy when loaded at low strain rate (at 10^−5^/s).
Pangolin scale (Al 6061-T6) by 3D Printing [226]	Ballistic experiment for armor. Projectile fired: Hard steel core Projectile mass: 0.785 kg Projectile velocity: 400 m/s Angle of attack: 0° Distance of attack: 10 m	Improved impact resistance through deflection of target due to the cantilever action (flexibility) offered by the single scale. Increased scales resulted in plugging of projectile due to absence of cantilever action.
Beetle wing-inspired honeycomb structure/column tubes (Aluminum) [239]	Impact velocity: 10 m/s Mass: 500 kg	More than 50% improved energy absorption under axial loading. Changing column thickness 0.5 mm–3.0 mm led to an increase in the total absorbed energy and energy absorptive effectiveness. Changing column height 50 mm–200 mm led to an increase in energy absorptive effectiveness first and then decrease due to buckling of the structure.
Oxhorn-inspired columnar tubes (Aluminum) [240]	Crashworthiness performance. Impact velocity: 10 m/s Mass: 500 kg	Specific energy absorption of bionic column was 46.2 kJ/kg and 1.3 times and 1.8 times higher than the four-cell conical tube. Energy absorption of bionic column increases with the increased thickness of the core.
Yakhorn-inspired columnar tubes (Aluminum foam) [241]	Loading rate: 5 mm/min Mass: 500 kg	Specific energy absorption of bionic tube was 51.23 kJ/kg and is 1.25, 0.89, and 1.02 times higher than that of circular tube, square tube, and tapered tube. Energy absorption capacity increases with the increase in taper, and then decreases.
Woodpecker’s beak-inspired honeycomb sandwiched structure (AA6060 Al-alloy) [231]	Impact velocity: 6–7 m/s Mass: 1 kg	Specific energy absorption of the bionic structure increases by 125% and 63.7%, respectively, compared with that of the honeycomb sandwich panel with the same thickness core or the same volume core.
Nacre-inspired hierarchical structure (Photopolymer based soft matrix staggered composite) by 3D printing [242]	Strain rate: 0.156 and 0.178 min^−1^ Cyclical tensile strain loading at 1 Hz	Tests conducted on polymer composites revealed superior damping performance, with a loss modulus reaching approximately 0.5 GPa, significantly surpassing that of individual polymers. The damping enhancement is attributed to the large shear deformation of the viscous soft matrix and the large strengthening effect from the rigid inclusion phase.
Nacre-inspired composite (Stiff material—PLA; soft matrix—Nylon and Thermoplastic Polyurethane) by Fused deposition modeling [243]	Impact velocity: 3.13 m/s Mass: 20.41 kg Impact energy: 100 J	A comparative study of two different materials for soft matrix was carried out. Drop impact tests showed that there were increases of 25% and 120% in the energy absorption capabilities of the structure. Dimensions of the tablets and thickness of matrix play a major role in structural properties.
Conch Shell-inspired hierarchical structure (Stiff material—Veromagenta Soft material— TangoBlackPlus) by 3D printing [244]	Impact velocity: 2–3 m/s Load: 2500–7000 N Impact energy: 13–25 J	Impact tests demonstrate tortuous stress paths and even distribution within the bioinspired structures, leading to increased energy dissipation. Second-level hierarchy structures exhibit 70% and 85% increases in impact performance compared to single-level hierarchy structures and the stiff constituent, respectively.
Cybister Elytra-inspired sandwiched structure (Ti6Al4V alloy) by SLM [245]	Multi-objective optimization	Optimizing bioinspired sandwich structures involve exploring various core layers and arrangements. Two-layered core structures with vertically arranged configurations exhibit excellent mechanical properties, specific energy absorption of 9.16 × 10^3^ J/kg, and energy absorption of 154.80 J.
Beetle Elytron-inspired sandwiched structure (Stainless Steel) by Direct metal laser sintering [246]	Tensile and compression experiment. Loading rate: 1 mm/min	The crash box test determined that the structure absorbed 375.5 J of energy, approximately 5 times more than the conventional structure.
Shrimp-inspired sandwiched structure (Photosensitive resin and nickel-coated carbon fibers) by Stereolithography 3D magnetic printer [112]	Compression and Charpy impact experiment. Loading rate: 1 mm/min	Magnetic 3D printing was utilized to fabricate Bouligand and herringbone architectures. The impact toughness and compression strength of the composites are contingent upon the orientation and distribution of the fibers. Bionic structures demonstrate enhanced fracture resistance and greater energy dissipation compared to traditional counterparts.
Turtle shell-inspired sandwiched structure (Photosensitive resin and nickel-coated carbon fibers) by Stereolithography 3D [247]	Tension and bending test. Loading rate: 2 mm/min and 0.15 mm/min. Impact wear test. Impact cycles: 10^3^, 10^4^, 10^5^ Impact velocity: 184 mm/s, i.e., Kinetic energy: 7.6 mJ Force: 100 N	Studied hydration effects (12 h, 24 h, and 48 h) on mechanical and impacting properties of turtle shell. Under the same impact cycles, energy absorption and contact time increased with the extending of soaking time. The absorption rate is the lowest for shell without soaking.
Bamboo-inspired honeycomb structure (Nylon/Carbon fiber) by 3D printing [237]	Quasistatic compression test. Force: 10 kN Velocity: 2 mm/min until a displacement of 40 mm	Bionic structure absorbed 755.83 ± 39.6 J of energy. Bamboo-inspired structure shows better energy absorption than snake- or beetle-inspired structures.
Balanus-inspired thin-walled tube structure (AISI 304L) by Dep drawing [248]	Dynamic crushing test. Impact velocity: 4 m/s Impact velocity: 0.025 mm/s	Bionic shell absorbed more energy throughout the deformation history. High load carrying capacity of bionic structure due to hard outer core and soft hierarchical inner core. Crushing force of the balanus structure increased by 26%.
Horsetail-inspired thin-walled tube honeycombs structure (Aluminum alloy) by Simulation study [249]	Axial dynamic loading of different cross-sectional configurations (i.e., number of cells) in the bionic structure. Impact punch mass: 1000 kg Impact velocity: 15 m/s	Structures with 16 cells are recommended as energy absorbers. 16 cells were found to have the best overall crashworthiness for vehicle body applications.
Pomelo-inspired hierarchically structure (Actual pomelo peel) [250]	Free Fall. Impact height: 10, 13.5, 18 m	10 m height potential energy between 83.4 and 98.1 J. No sign of damage found. 13.5 m height potential energy between 100 and 117.9 J. No sign of damage found. 18 m height potential energy between 117 and 158.9 J. No sign of damage found.
Pomelo-inspired hierarchically structure (PEEK) by 3D printing [236]	Compression test. Loading rate: 0.02 mm/s Compression strain: up to 70%.	Guidance to design lightweight materials with high energy absorption. Crush force efficiency and dropping force efficiency are closer to 1 as compared with the non-pomelo peel-inspired structures. No abrupt changes in compression forces for bionic pomelo PEEK structures.
Cashew nutshell-inspired biocomposites (ABS/Hemp/Glass fiber) by 3D printing [238]	Tensile, Flexural, Compression, Izod impact, Low velocity drop load impact, Fatigue, Fracture toughness tests.	Best composition in vol.%: Resin: 60; Fiber: 30; ABS: 10; Lignin: 1 Maximum mechanical property achieved: tensile strength, flexural strength, Izod impact, and compression of 136 MPa, 168 MPa, 4.82 kJ/m^2^, and 155 MPa, respectively. Maximum fatigue life counts noted for the composite about 33,709, 25,781, and 19,633 for 50%, 70%, and 90%. Maximum fracture toughness compared to pure epoxy resin, with value of 32.5 MPa·m^1/2^. Highly toughened and marginally flexible composites could be potentially employed in the fabrication of high-endurance morphing wings for unmanned aerial vehicles and aircraft wings.
Turtle shell-inspired sandwiched structure (Rubber/Carbon fibers) by Vacuum assisted resin injection [49]	Drop hammer impact test. Impact mass: 7.29 kg Impact energy: 30 J, 90 J Impact height: 0.42 m, 1.26 m	Turtle-inspired structures show an improvement in impact resistance by 10–25%. Main impact failure modes include coating fracture, fiber fracture, compression damage deformation failure of the rubber core, fiber delamination, and interlayer degumming. Size of the hammer head radius has a significant effect on the energy absorption characteristics. Hammer head radius is 10 mm; the absorbed energy and specific energy absorption increase by 25% compared with hammer head radius of 8 mm and 83%.

**Table 6:** Results of various bioinspired designs exploited for the enhancement of cutting or machining tools, as reported in the literature.

Bioinspired Structure	Conclusions/Texture Effect
Sea urchin teeth-inspired rock-cutting tool (Crystalline calcite teeth/epoxy composite) [271,272]	Self-breaking or fracturing leads to self-sharpening. Detailed description of self-sharpening phenomena.
Dung beetle-inspired Bulldozing plate (UHMWPE) [267]	Bionic UHMWPE could reduce soil adhesion and draught considerably. 30% reduction in cutting resistance.
Lotus leaf/Shark skin-inspired soil shovel/tillage tool (Steel-based) [266]	1.85–4% reduction in cutting resistance.
Dung beetle-inspired toothed wheel tool (Steel-based) [35]	16.5% reduction in cutting resistance. 11.8% reduction in stress concentration. 24% increased microbasin.
Shark skin-inspired subsoiler tillage tool (Steel-based) [303]	21.9% reduction in cutting resistance. 24.8% reduction in consumption energy. Improved crop stress resistance.
Mole pelt-inspired cutting blade for tillage tool (Steel-based) [36,282]	12.8% reduction in cutting resistance. 4% reduction in torque. 60% improved stubble breaking rate.
Pangolin-inspired drill bit for dry rock cutting (Impregnated diamond bit) [260]	97.5% increased drilling speed. 26.8% increased service life.
Mole claw-inspired drill bit (Impregnated diamond bit) [264]	230% increased drilling efficiency. 345% increased service life.
Beetle/Earthworm-inspired drill bit (Impregnated diamond bit) [191,261]	91% increased wear resistance. 27% increased grinding performance.
Bamboo/human tooth/annual ring/shell/mole pelt-inspired drill bit (Impregnated diamond bit) [285]	Improved cutting resistance. Improved wear resistance. 250% increased penetration rate.
Cat claw-inspired drill bit (Impregnated diamond bit) [304]	13% increased penetration rate.
Dung beetle-inspired drill bit (cemented carbide bit) [41]	45% increased drilling speed. 23% decreased wear rate.
Dung beetle-inspired microgrooves/pits turning tool for dry cutting (AISI 1045 steel) [305]	24% decreased cutting force. 29% decreased cutting temperature. Grooves and pits led to wear debris (graphite) storage and improved lubrication.
Dung beetle-inspired microgrooved tool for dry machining of Ti-alloy (PCD tools) byFiber lasers [278]	Reduced friction coefficient. Reduced tool-chip contact length. Reduced cutting force. Reduced adhesion.
Rat claw-inspired microdimples/grooves for dry cutting of Ti-alloy (cemented carbide tool) [296]	Increased tool’s anti-adhesion ability. Increased chip-breaking efficiency. Increased chip curling.
Snakeskin-inspired tool for dry cutting of AISI/SAE 4140 (cemented carbide inserts) byFemto second lasers [294]	65% reduced cutting temperature. 51% reduced cutting power. 16% reduced cutting force. Reduced sticking sliding contact.
Sea urchin/Shark teeth-inspired microtexture/serrated cutting edges for finish turning of ferrous alloys. (PCBN inserts) [44]	Reduced tool wear. Reduced finished surface roughness, Ra.
Corn leaf’s teeth-inspired microgrooved blade for milling of Al-alloy byFEM simulation [289]	10% reduced cutting force. Reduced cutting temperature. Reduced cutting vibration.
Beetle-inspired microtexture on tool cutter for milling of Ti-alloy (PCD and carbide tool) [306]	7% reduced cutting stress. Reduced cutting force. Reduced tool wear. Reduced cutting force fluctuation.
Badger teeth-inspired microdimples/grooves on circular saw blade (Steel-based tool) [307]	Reduced cutting torque. Reduced workpiece tension. Increased cutting quality.
Shark skin-inspired microgrooves on cutting tool with WS_2_/C coating (Carbide tool) [298]	Reduced flank wear. Reduced surface roughness. Reduced surface temperature.
Desert scorpion-inspired microgrooves on cutting tool (Carbide tool) [299]	Reduced flank wear. No effect on surface roughness. Textures on the flank faces could also act as storage for powder chip and can protect the surface from abrasion.
Scorpion or dung beetle-inspired microgrooves or dimples on cutting tool (Carbide tool) byLaser processing [293]	4.2% reduction in friction in dry cutting condition. 5.1% reduction in friction in oil lubrication. Superior chip flow in groove microtexture perpendicular. Groove microtexture can store lubrication oil and improve lubrication and cooling effect during cutting.

## Data Availability

Some or all of the data, and models that support the findings of this study are available from the corresponding author upon request.
